# Development of near real-time wireless image sequence streaming cloud using Apache Kafka for road traffic monitoring application

**DOI:** 10.1371/journal.pone.0264923

**Published:** 2022-03-17

**Authors:** Aung Myo Htut, Chaodit Aswakul

**Affiliations:** Wireless Network and Future Internet Research Unit, Department of Electrical Engineering, Faculty of Engineering, Chulalongkorn University, Bangkok, Thailand; Al Mansour University College-Baghdad-Iraq, IRAQ

## Abstract

In this paper, the authors have designed and implemented the prototype for a near real-time wireless image sequence streaming cloud with two-layered restoration for a road traffic monitoring application of a small-scale network. Since the proposed design is targeted to implement outdoors where the link or node failure could occur, the fault-tolerant capability must be considered. Having only one layer restoration may not provide a good quality of service. Therefore, a two-layer restoration framework is designed in the proposed system by restoring the network layer with the underlying software-defined wireless mesh network capability and at the local broker selection over the Apache Kafka framework. The monitoring application performance has been investigated for the end-to-end average latency and image loss percentage by outdoor testing for 13 hours from 5:40 P.M. 17^th^ November 2020 to 6:40 A.M. 18^th^ November 2020. The end-to-end average latency and image loss percentage have been found to be within the acceptable condition i.e. less than 5 seconds on average with approximated 10% image losses. The proposed system has also been compared with the traditional ad-hoc network, running the OLSR-based network layer, in terms of the rerouting time, restoration time and end-to-end average latency. Based on the emulated wireless network in controllable laboratory environments, the proposed SDWMN-based system outperforms the conventional OLSR-based system with potentially faster rerouting/restoration time due to SDN central controllability and with only marginally increased end-to-end average latency after re-routing/restoration completion. Algorithm complexity analysis has also been given for both the systems. Both the experimental and complexity analysis results thus suggest the practical applicability of the proposed system. Given this promising result, it is therefore recommended as the future research in further developing from the prototype design into the actual deployment for daily traffic monitoring operations.

## Introduction

Bangkok has been recorded as the tenth-ranking most congested road traffic condition globally [1]. The main reasons for that problem are the insufficient road capacity, and the random events (abnormal events) that can cause the increasing traffic flow [2, 3]. Due to network complexities, automatic controls of traffic signal light become ineffective. This is particularly evident in developing countries where necessary control sensors are hardly available. By trials-and-errors, the problem of short-term traffic flow management must be tackled. In Bangkok, like many other big cities, traffic police can mitigate the congestions by controlling the traffic signal light only when they know the real-time or near real-time road traffic network information [4].

In practice, having equipment for road traffic monitoring can give advantages, i.e. reducing risks [5]. To have the traffic monitoring system (TMS), the camera sensor has the advantage for the traffic police to evaluate, to understand and really to know the actual road traffic condition. Camera sensors also play an essential role in analysing the data in intelligent transportation systems (ITS).

To use the camera sensor nodes, nowadays, optical fiber is often used for closed-circuit television (CCTV) surveillance monitoring systems. This CCTV surveillance monitoring systems is the current standard practice in many cities. As a result, the losses and the latency for video streaming are low. However, there are challenges in such standard CCTV deployment when one needs to send the images or video from the roadside camera to the traffic police controller box where traffic police can control traffic signal lights. The main challenge is the service deployment cost, which is high because of the very long-distance wired communication line installation.

To reduce the cost of the wired connection, a wireless connection has been recently adopted worldwide. With expected cost reduction, however, the service experience in terms of loss and latency must still be provisioned within the acceptable condition. To satisfy with the desired service experience, one solution is streaming the video to the control center via the 4G Internet connection [5]. However, using only 4G Internet connection directly from every camera is not cost-effective. Therefore, the hybrid wireless connection, both Wi-Fi and 4G Internet connection, has been investigated. However, for the hybrid wireless connection with the installation of the medium-range distance (with per-hop coverage of 200 to 300 meters), there are other challenges. The main challenges are low quality of service (low throughput and low bandwidth) and randomly occurring wireless line-of-sight blockage. Because of these challenges, achieving a cost-effect wireless ad-hoc network streaming application with a satisfactory service experience becomes non-trivial. With the Wi-Fi connection, throughput is low, and each hop with wireless line-of-sight between ad-hoc network nodes can be temporally randomly blocked. Therefore, there is a need for effective re-routing, referred in this paper as the lower-layer network restoration. However, in serious physical network decomposition events, such network rerouting would at times become impossible. Therefore, in this paper, another restoration mechanism at the application layer running the Apache Kafka [6] message brokerage has been proposed. The resultant mechanism forms the so-called two-layered restoration framework i.e. at the network layer and the application layer.

The overall design is as follows. To reduce the cost, a cost-effective computing board, i.e., Raspberry Pi and Raspberry Pi camera, have been used as the image sending node. Road traffic images are sent periodically from Raspberry Pi’s to an adaptively selectable local broker at the nearest local traffic police controller box with Apache Kafka (hereinafter called Kafka) framework [6]. Incoming image sequences are also forwarded to the traffic data cloud, enabling the traffic police command center to monitor the overall road traffic conditions and for the local traffic police at the neighboring areas to pull relevant traffic data for operating area-coordinated traffic controls. External broker at the traffic data cloud and local brokers at local traffic police controller boxes store the log file for future data analysis and also subsequent data pulling requests.

In the remainder, this paper reports on the real-world prototype implementation of near real-time wireless image sequence streaming cloud using the Apache Kafka for the road traffic monitoring application. At the application layer, an automatic switching mechanism has been proposed to select the best Kafka broker for image streaming. Such automation would be triggered upon the failure to restore the current primary best path of operations at the underlay network layer. Additionally, at the network restoration layer, the concept of software-defined networking has been deployed to develop a sufficiently fault-tolerant system for near real-time usage, and this system must achieve an end-to-end streamed image sequence delivery to the target users. The fault-tolerance concern is because of the node or link failure, which results in the images not arriving successfully at the nearest local traffic police controller box. With the constructed outdoor testbed, and the constructed laboratory-based emulated testbed, the proposed system has then been thoroughly evaluated in this paper.

## Background and related work

### Software-Defined wireless mesh network

**Fig 1:**
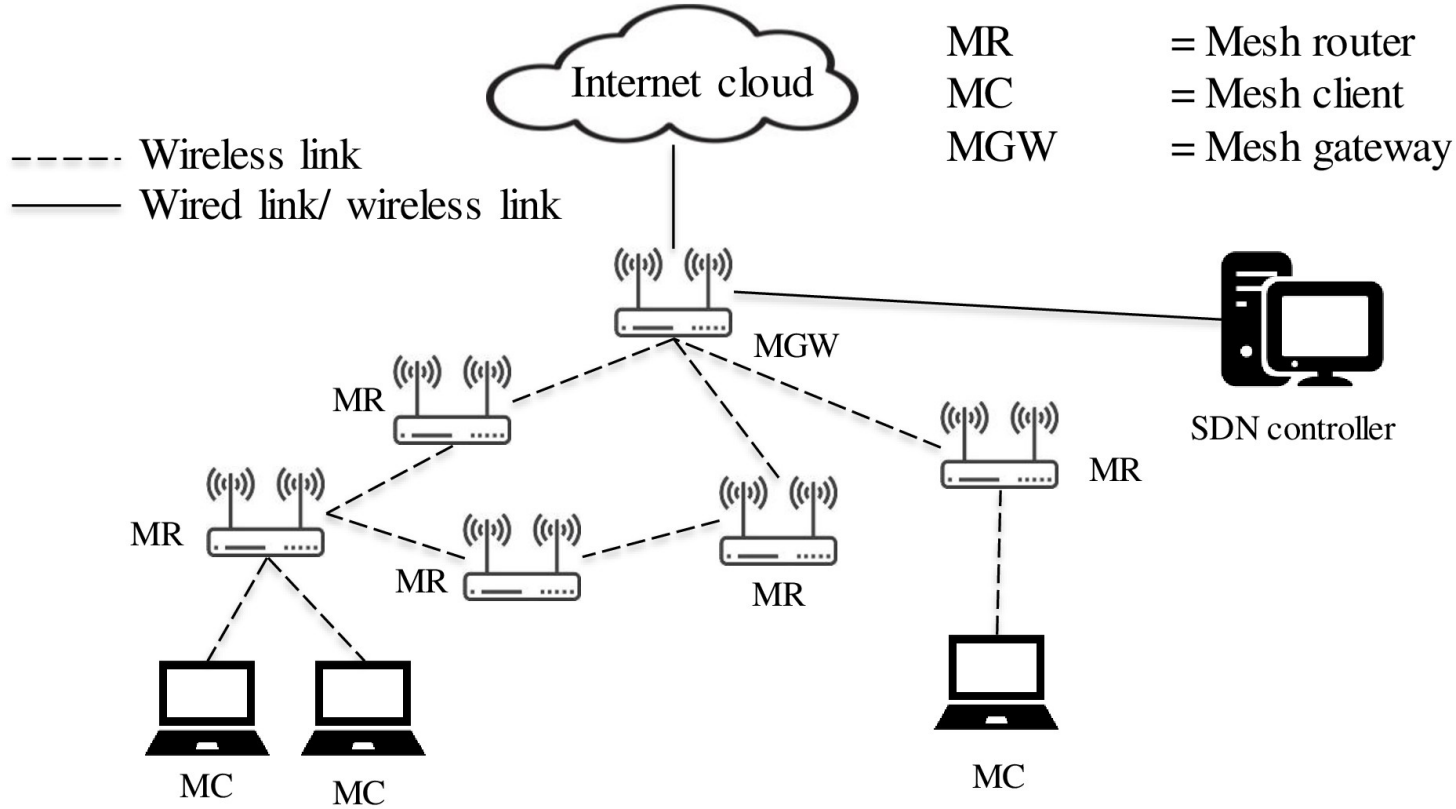
Typical architecture of SDWMN [7, 8].

As mentioned in Section “Introduction,” to reduce the cost for the communication medium, in this work, the wireless mesh network (WMN) is used. However, the traditional WMN has limitations at the centralized control layer. To solve that limitation, there are emerging technologies such as software-defined networking (SDN). The concepts of SDN and WMN are merged in [7, 8] and the result is Openflow enabled software-defined wireless network (SDWMN). This network must be designed by considering topology robustness with self-healing characteristics as enabled by a proper mesh network routing using the enhanced network programmability. A typical type of SDWMN network is demonstrated in Fig 1 SDN controller is added in the traditional WMN to control the data routing from each mesh node to the mesh gateway.

### Apache Kafka distributed messaging system

**Fig 2:**
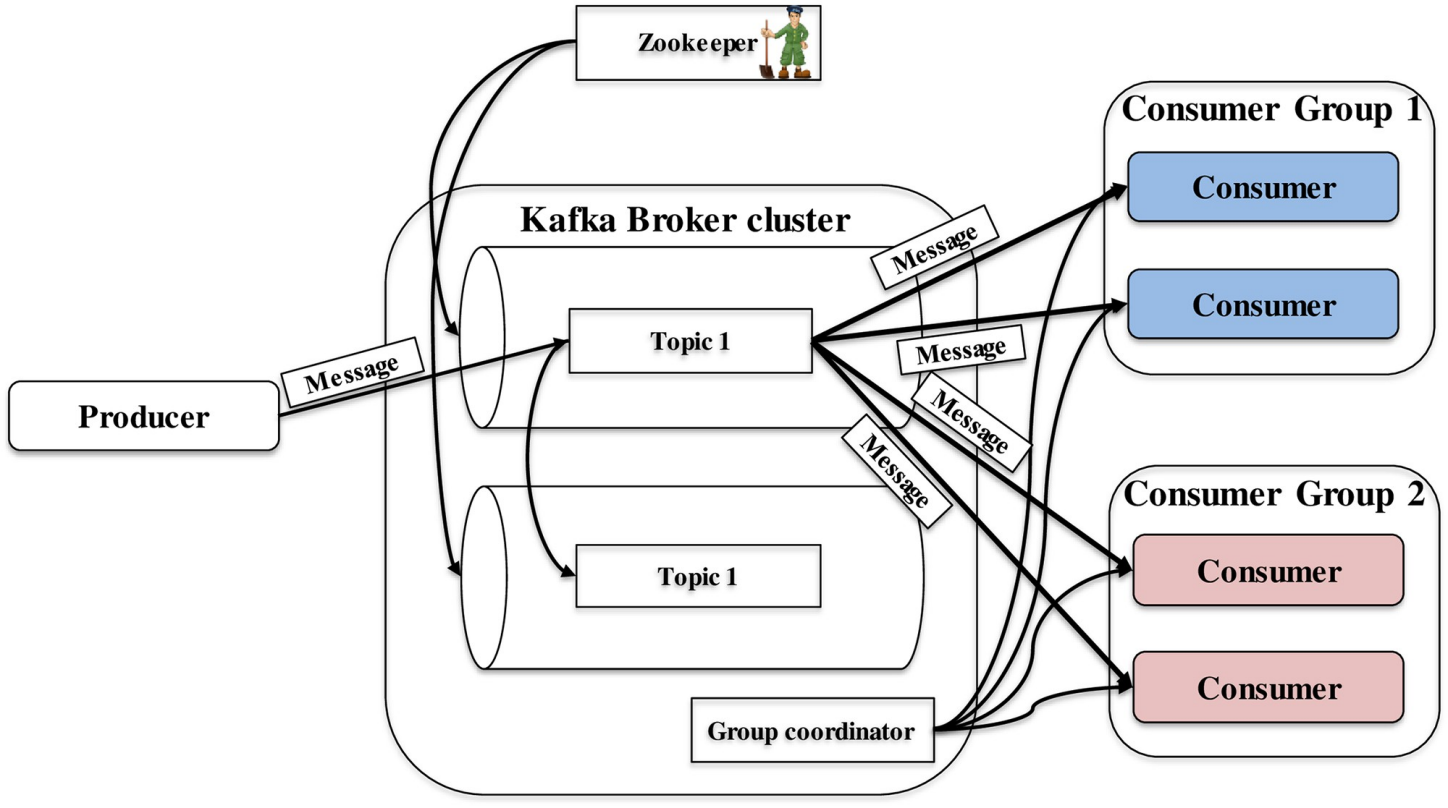
Typical architecture of Kafka [6].

Kafka is designed to have high-throughput, and low-latency [6]. The typical architecture of Kafka is shown in Fig 2.

Kafka can provide the delivery guarantee [9]. In the delivery, there are three variants as follows:

- “At most once” does not guarantee a successful delivery because the message is sent to the broker, not more than once. The producer does not care whether the sent message is successfully stored inside the broker or not. Therefore, there is no guarantee that the sent messages to be successfully stored by the broker. This kind of streaming message can be useful when the message loss is not essential, and the transmission delay is needed to be low enough.
- “At least once” delivery guarantee ensures that every sent message is stored at least once at every device in the cluster. The duplication of received and stored messages can occur due to the message re-transmission by a sender when the sender receives too late message reply acknowledgment. Therefore, messages are stored at every broker successfully. This variant can guarantee no losses. However, this variant has the disadvantage that the message sender needs time to get the reply message from every intended receiver device.
- “Exactly once” delivery guarantee ensures that the intended receiving device successfully stores the incoming message. The delivery is guaranteed for each message to be stored successfully exactly once, even when the received message duplication occurs.

In this work, “at most once” and “exactly once” are chosen for streaming the images because the successful image storage inside the broker and a slight transmission delay are essential to send the images from the cameras to the traffic police controller boxes. Messages are sent in batches. Within each batch, image messages are sent in “at most once” mode to reduce the transmission delay except the last message sent with“exactly once” mode to check whether the messages are arriving at the broker.

Apart from the delivery guarantee, Kafka has long-term message storage as the Kafka messages are stored at the local storage system. Kafka and other systems can have the reliable streaming of data via a framework called Kafka connect. For stream processing, Kafka steam can be used. Because of these Kafka features, we decided to use the Kafka framework in our proposed TMS.

### Related work on data sources and communication technologies in existing TMS

In addition to the intended near real-time road TMS in this work, the system needs to be implemented along the targeted congested road. In this regard, the past TMS approaches have first been reviewed in Table 1 to understand state-of-the-art.

**Table 1:** Summary of reviewed data sources and communication link using in TMS.

Paper	Real-time or near real-time	Communication link	Data type	Mobile or fixed
[16]	Yes	3.5G/WiMAX	In-car video and roadside videos	Mobile
[17]	Yes	Optical fiber 1 Gigabit Ethernet	384 x 288 resolution MPEG-4 format video	Fixed
[12]	Yes	GPRS and GSM	GPS on the bus	Mobile
[13]	Yes	ZigBee and GSM	Probe vehicle with RFID tag	RFID reader is fixed
[14]	Yes	Wireless sensor network	Probe vehicle with RFID tag and GPS	RFID reader is fixed
[11]	Not mentioned	ZigBee	Magnetic sensor	Fixed
[10]	Yes	Wireless sensor network and Internet	Ultrasonic sensors	Mobile
[15]	Not mentioned	Bluetooth mobile ad-hoc network	Probe vehicle with Bluetooth device	Mobile
[19]	No	Not mentioned	Snapshot image	Fixed
[18]	Yes	Wi-Fi with backbone optical network and WiMAX with optical backbone network	Low frame rate video	Fixed

In Table 1, there are two categories of sensor data types. In the first category, the sensor returns numerical output results e.g. vehicle counted number for each lane [10], magnetic field intensity value to detect vehicle presence [11], road density in terms of traffic flow rate [12], average velocity for each lane [13], number of vehicles inside the sensing area [14] and surrounding vehicle detection with Bluetooth [15]. These outputs are most useful for statistical data analytics that would give insightful strategies in managing traffic flows on the road. However, for daily operations of traffic flow management by traffic police, such numerical output results from the sensor nodes are usually too difficult to understand especially for the busy on-duty traffic police who control the traffic signal light. Therefore, these sensors become unuseful for the traffic police who would like to watch the actual condition of the road traffic rather by themselves with their own eyes. In the second category, the sensor returns directly the video or image of surrounding road traffic conditions. This is also the main focus of this paper concerning video or image streaming. In this regard, the past works include e.g. [16–18]. In [16], road-side video and in-car videos have been delivered with 3.5G/WiMAX. In [17], the use of optical fiber to stream MPEG-4 videos from 600 cameras has been introduced in Valencia, Spain. The video quality and resolution are good by using optical fiber since video can be streamed on the high throughput link. Work in [18] reports that the low frame-rate video enough should be sufficient for the traffic police to know the road traffic condition. The work has also compared the system between the Wi-Fi with backbone optical network and WiMAX with backbone optical network. These wireless systems with a backbone optical network would also need a high installation, operation, and maintenance cost.

The main difference between our work and those summarized works with video-sensor type is that we propose the near real-time wireless image sequence streaming cloud with the network-layer rerouting that is combined together with the application layer of local broker re-selecting application for TMS application. In this testbed, SDWMN developed by the author in [20] is used as a network layer for streaming the image sequences of the road traffic from the Raspberry Pi’s. On top of the network layer, the Apache Kafka is used at the application layer for streaming, storing, and playing back the captured traffic image sequences to the traffic police. To the best of our knowledge, none of the past works in Table 1 has investigated the usage of restoration mechanisms in both the network layer and the application layer before.

## Proposed simple fault-tolerant near real-time wireless image sequence streaming cloud

The proposed architecture is designed on foundations obtained previously from the preliminary testbed designs in [21]. To cover the road segment of the Phaya Thai road between Rama 1 road and Rama 4 road and to capture both movement directions of the road, more cameras are needed to be installed. A 1km length of the road segment is tested to stream the image sequences to develop a prototype. To test on the 1km of road length, the Phaya Thai road segment between Rama 4 and Chulalongkorn Soi 12 are chosen.

### Design of underlying SDWMN in-band architecture

The network layer for the proposed architecture is based on the network architecture designed in [20]. SDWMN in-band architecture is used in this testbed, and the Ryu controller is located in the cloud. Two separated devices are used at the traffic data cloud, one for the Ryu controller and one for the external broker to have a clear point of view and control. SoftEther virtual private network (VPN) [22] is used to connect between the local brokers and the traffic data cloud. The two local brokers are connected to the Ryu controller via the VPN bridges. The rest mesh nodes are connected to the Ryu controller via the local brokers to be the in-band architecture. The detailed information for the underlying SDWMN network is explained in [20].

### Design of interworking between application-layer and SDWMN

**Fig 3:**
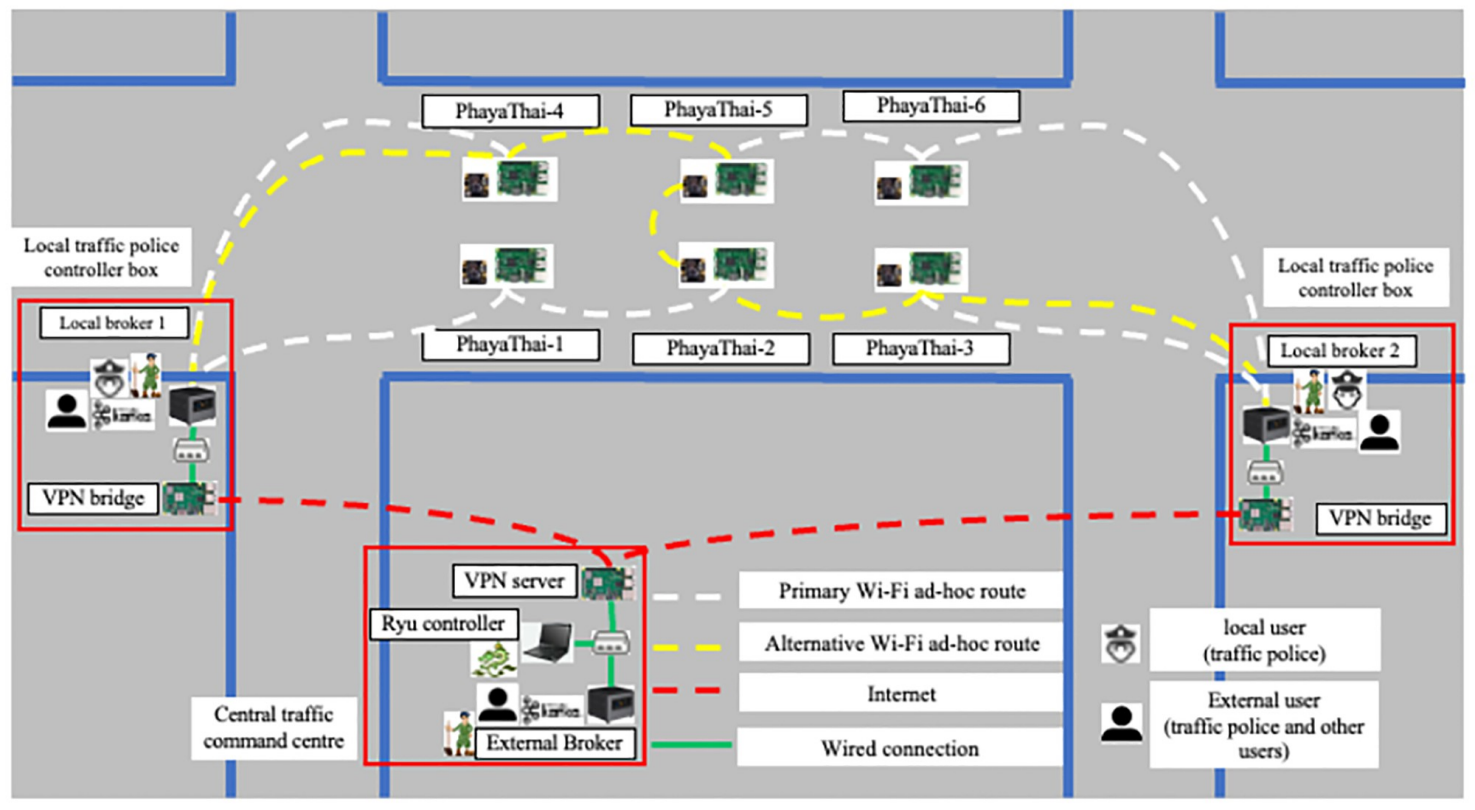
Proposed architecture for road traffic morning application.

The proposed architecture includes cameras, local brokers, an external broker, and a Ryu controller, as shown in Fig 3. To have a clear view for the traffic police user and the proper networking in the SDWMN network, Raspberry Pi’s are installed at the fence of the crossover bridges of Phaya Thai Road, Bangkok.

At the physical locations of the target testbed, which is the Phaya Thai road segment near Chulalongkorn University, Bangkok, the actual distance is from 200 to 300 meters in-between adjacent crossover bridges. Therefore, the per-hop wireless ad-hoc reachable distance must be between the adjacent two crossover bridges and the distance from the crossover bridge to the nearest traffic police controller box. With this distance, traffic police can know the actual condition of the Phaya Thai road segment. The location of local broker 1 is at the junction of the Phaya Thai road and Rama 4 road. Local broker 2 is at the intersection of the Phaya Thai road and Chulalongkorn soi 12. Both locations are chosen because the distance between the local brokers and the nearest Raspberry Pi’s is 200 to 300 meters apart. This distance is also met with the per-hop wireless ad-hoc reachable distance. Traffic police in those locations play critical roles in controlling the road traffic flows along the Phaya Thai road and other neighbor road segments.

Certainly, for other cities with a relatively longer stretch of road than in our scenario, we believe our proposed design is directly extensible at the physical layer, e.g. increasing the antenna amplification gain or directionality, or at the network-layer simply by installing SDN flow rules to accommodate needed route plans.

Six mesh nodes are separated into two groups according to their distance to reach the nearest local traffic police controller boxes as shown in Fig 3. Mesh node names are given according to the location of the camera. For PhayaThai-1, 2, and 4, the default local broker is local broker 1. For PhayaThai-3, 5, and 6, the default local broker is local broker 2. Each mesh node publishes captured-image messages to the local broker with the Kafka topic, defined as the sending mesh node’s name. Based on the result of [21], 320 × 180 resolution JPEG image is chosen since the latency is acceptable with the smallest image size and the view is clear enough for traffic police.

Intel^®^ NUC7i7BNHs are used as local brokers, and each of the local brokers is installed at the local traffic police controller boxes. In the local traffic police controller box, a Kafka broker receives the incoming Kafka messages from the mesh nodes. The Kafka consumers show the incoming Kafka messages as the image sequences to the traffic police. Another Kafka producer forwards the incoming Kafka messages from the mesh node to the external broker with the same topic name.

The external broker, Ryu controller, and VPN server are installed at the telecommunication system research laboratory, engineering building 4, faculty of engineering, Chulalongkorn University. The external broker receives the forwarded Kafka messages from the local brokers, and these messages are stored as a log file for each topic. Ryu controller assigns the flow rule of mesh nodes. VPN server serves the VPN service to connect between the Ryu controller and local brokers.

**Fig 4:**
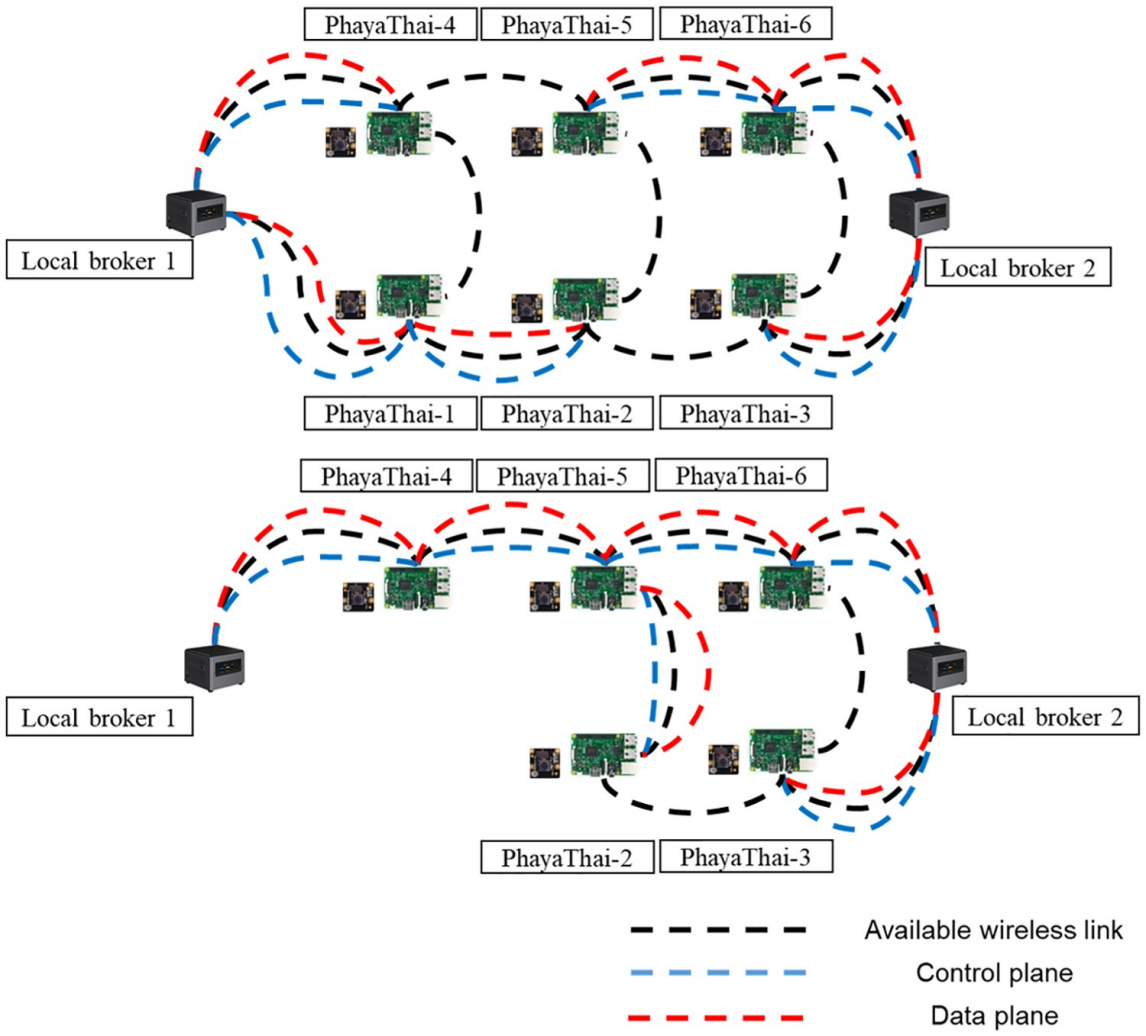
PhayaThai-1 unreachable case.

**Fig 5:**
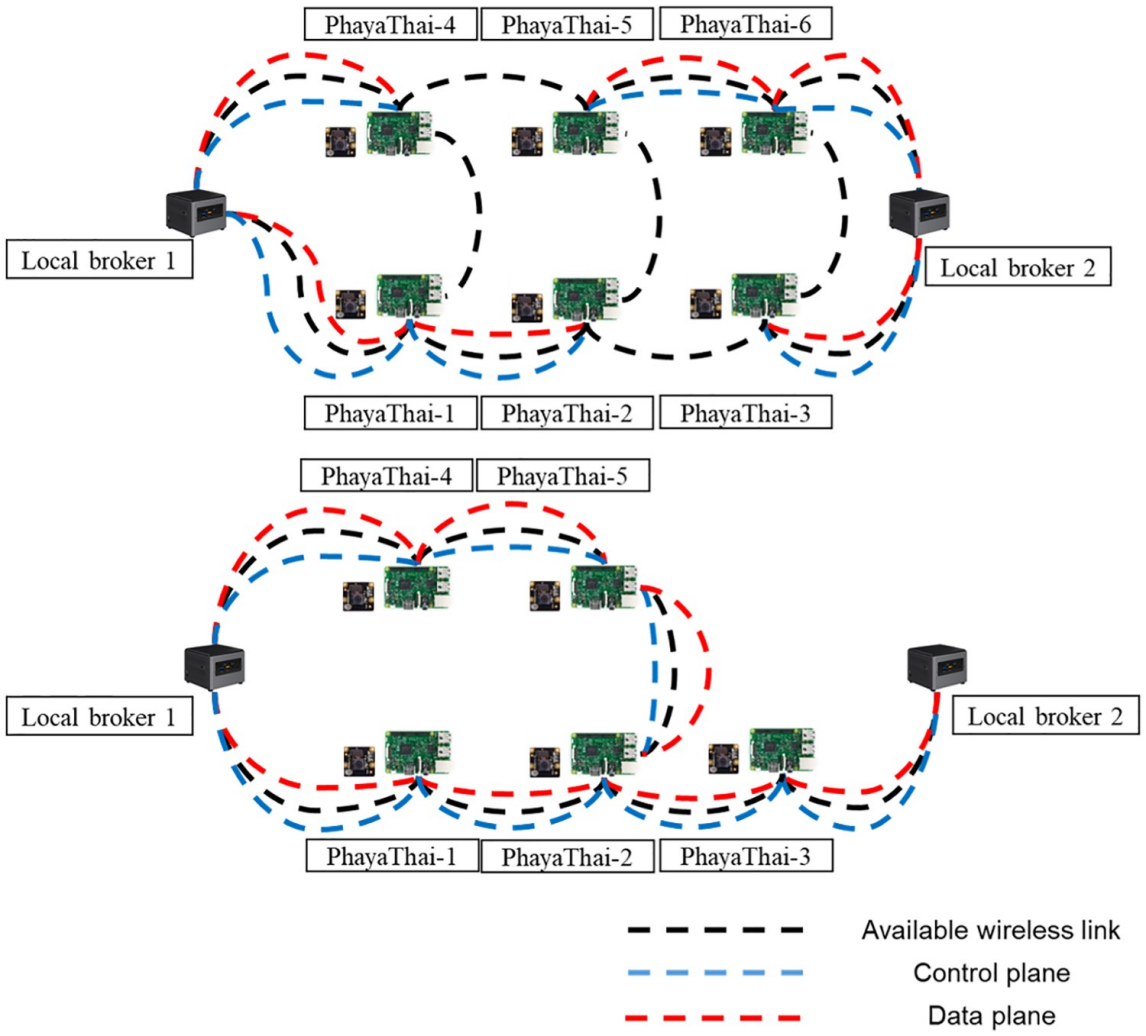
PhayaThai-6 unreachable case.

For the networking part, mesh nodes and local brokers are connected with the SDWMN network. Based on the SDWMN network test by authors in [7], mesh nodes are network-unreachable at times. When that happens, SDWMN will try to restore the network with the pre-planned re-routing scenario as shown e.g. in Fig 3, when PhayaThai-1 and PhayaThai-6 nodes are inoperable. If PhayaThai-1 node is unreachable, then PhayaThai-2 node will be re-routed to local broker 1 with the alternative route (PhayaThai-2—PhayaThai-5—PhayaThai-4—local broker 1) as shown in Fig 4. Likewise, if PhayaThai-6 node is unreachable, then PhayaThai-5 node will be re-routed to local broker 2 with the alternative route (PhayaThai-5—PhayaThai-2—PhayaThai-3—local broker 2) as shown in Fig 5. The Ryu controller periodically checks whether the mesh nodes are still alive. When the Ryu controller cannot get the reply message from the mesh nodes, the re-routing scenario occurs to re-connect all reachable mesh nodes to the default local brokers. This re-routing scenario needs a few seconds to check and re-assign the Open vSwitch (OVS) flow rule inside the mesh nodes. During the re-routing attempts of SDWMN in the network layer, the Kafka producer application at each mesh node would wait and not attempt any application-layer restoration mechanism. To solve this problem, Kafka producer at PhayaThai-2 and 5 can have a local broker switching mechanism at application-layer, and the detail is explained in the next Section.

Local brokers to the external broker have been connected via the VPN. Each local broker connects to one VPN bridge, and therefore the local broker can communicate with the external broker, which is the same network as the Ryu controller. VPN bridge is attached with a 4G wireless modern to use 4G Internet connection via a public telecommunication service provider.

### Operational flow charts of Kafka producer and consumer applications in simple fault-tolerant near real-time wireless image sequence streaming cloud

**Fig 6:**
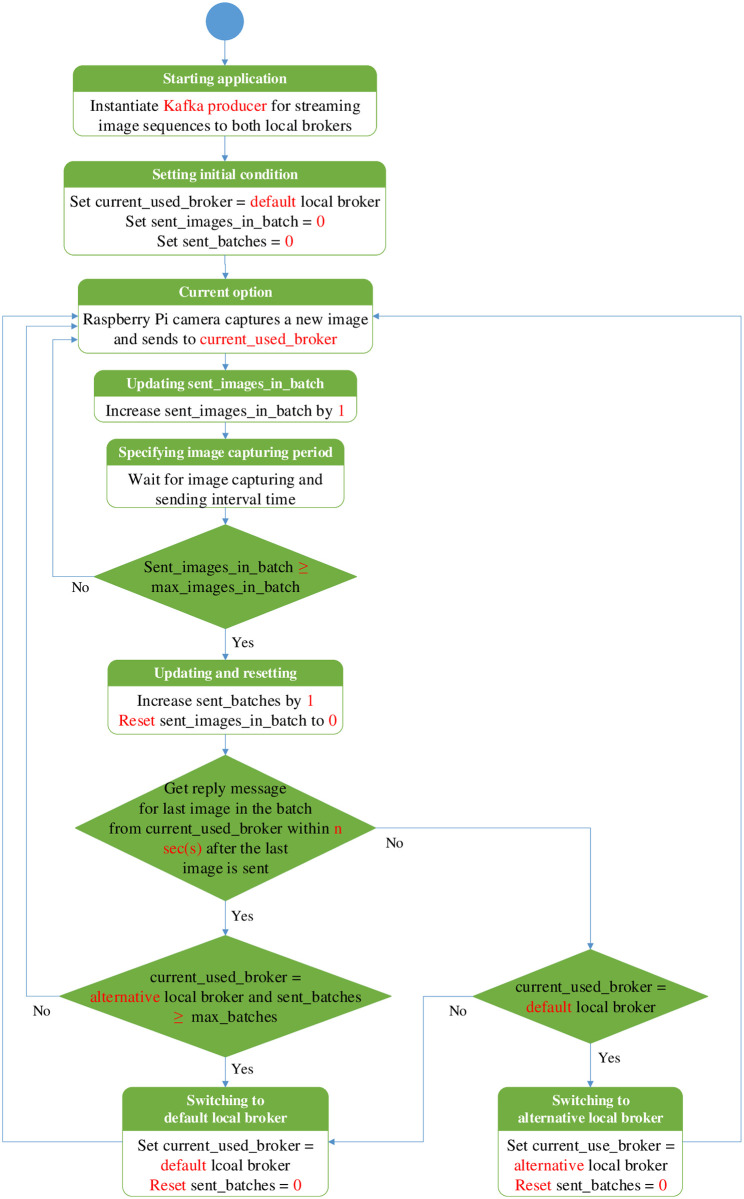
Operational flow chart of Kafka producer application with local broker switching mechanism inside the mesh node.

The flow chart of publishing the images as Kafka messages from PhayaThai-2 and 5 to the local brokers is shown in Fig 6. Mesh node first instantiates the Kafka producer for both local brokers. Raspberry Pi assigns the initial condition as explained in Fig 6. Raspberry Pi camera captures a new image and sends it to the current_used_broker with the Kafka topic defined as the name of that mesh node, e.g., PhayaThai-1. A new image is sent to that local broker periodically from the mesh node until the sent_images_in_batch reaches max_images_in_batch. After sending the last image in the batch, the mesh node waits for at most n sec(s) to get the reply message from the current_used_broker confirming that the last image in the batch is successfully stored at the local storage system of current_used_broker. To reduce the processing time in waiting for the reply message from the current_used_broker, to confirm successful delivery of message batch transmission, the mesh node waits for only the reply message of the last image in the batch. If the mesh node does not receive the reply message from the current_used_broker within a maximum threshold for waiting time n sec(s), then the mesh node switches to another local broker. Subsequently, Raspberry Pi camera captures a new image sequence. It sends the newly captured sequence with the number of images of max_images_in_batch to another local broker instead of the current one with the same one Kafka topic name. Since the default local broker is nearest to the mesh node, we limit the number of batches that can be used for the alternative local broker to max_batches. If the sent_batches reach max_batches, the algorithm will force the mesh node to switch back to the default local broker. At this time, if the wireless link or mesh node between the Raspberry Pi and the default local broker has already been restored, then the mesh node will continue subsequently sending to the default local broker. Otherwise, after losing one batch, the mesh node will switch back to the alternative local broker. Sending to the default local broker is the best way because that default local broker is the nearest local broker for the mesh node.

At each local traffic police controller box, the local broker is used with three functions. The first one is to serve as a local Kafka broker that receives the incoming Kafka messages from mesh nodes. The second function serves as a Kafka consumer from both the local Kafka broker and external Kafka broker. The third function is to relay the locally received messages from the local Kafka broker to the external broker.

**Fig 7:**
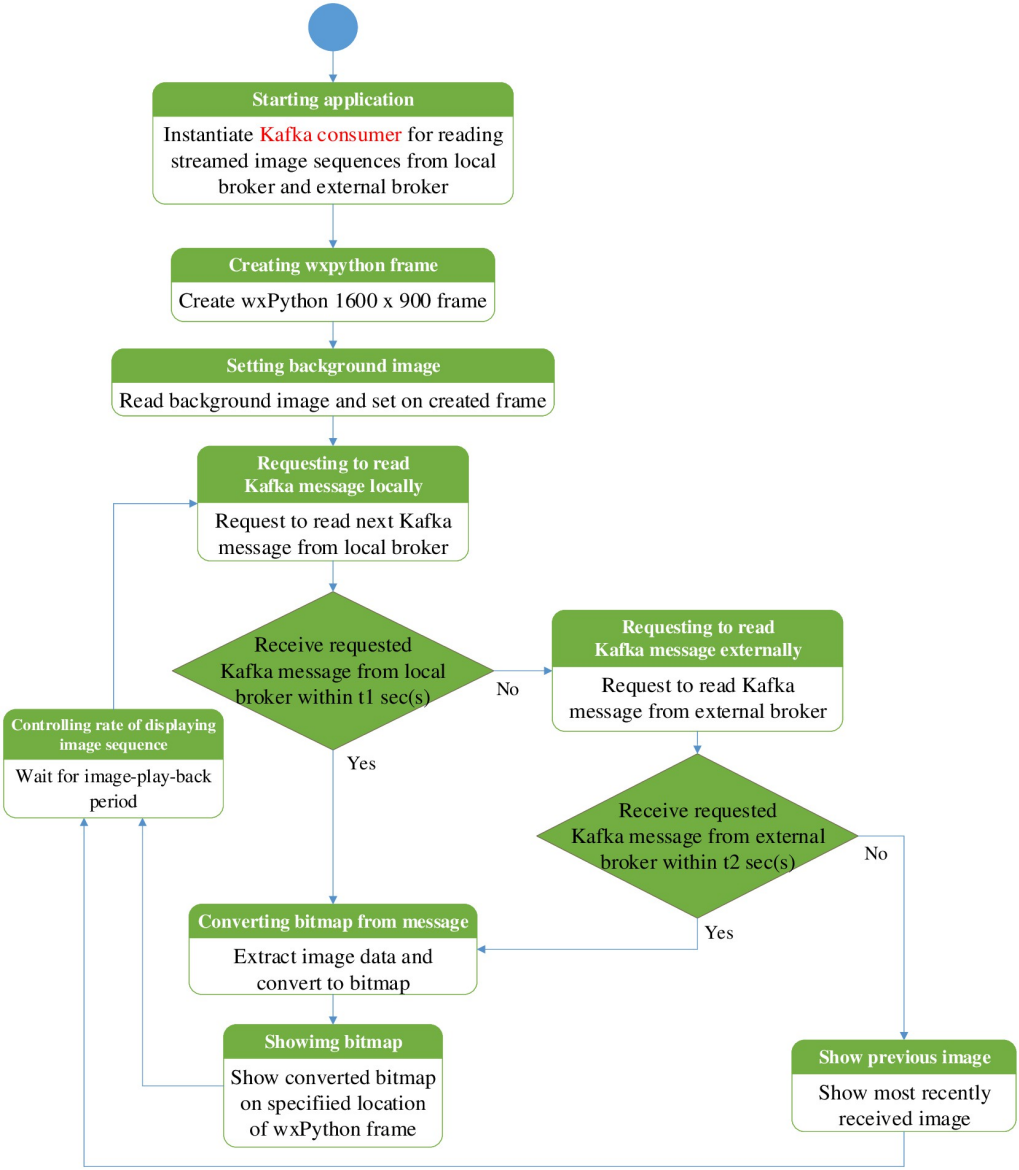
Operational flow chart of Kafka consumer application in local traffic controller box.

The second function here is depicted in Fig 7. At first, the monitoring application instantiates the Kafka consumers for the local brokers. After that, the wxPython frame is created with the pre-configured resolution of the monitoring screen. A background image is read from the local storage and shown on that wxPython frame. Multi-threading is used for reading and showing image sequences from all the Kafka topics published by mesh nodes. Each thread is used to read and display the messages in parallel from each Kafka topic. The image message is shown at the specified location for that camera location of the created wxPython frame. Suppose the monitoring application does not get the reply message from the local broker for the requested Kafka message within t1 sec(s). In that case, the monitoring application will switch to the external broker. Likewise, in the local broker, the monitoring application waits for t2 sec(s) to read the last incoming message from the external broker. t2 should be greater than t1 because a longer time is needed to connect to the external broker than to the local broker. If the monitoring application cannot find the new incoming Kafka message, the last received image is displayed instead at the created wxPython frame. This process of reading and displaying images is repeated for all subsequent messages.

**Fig 8:**
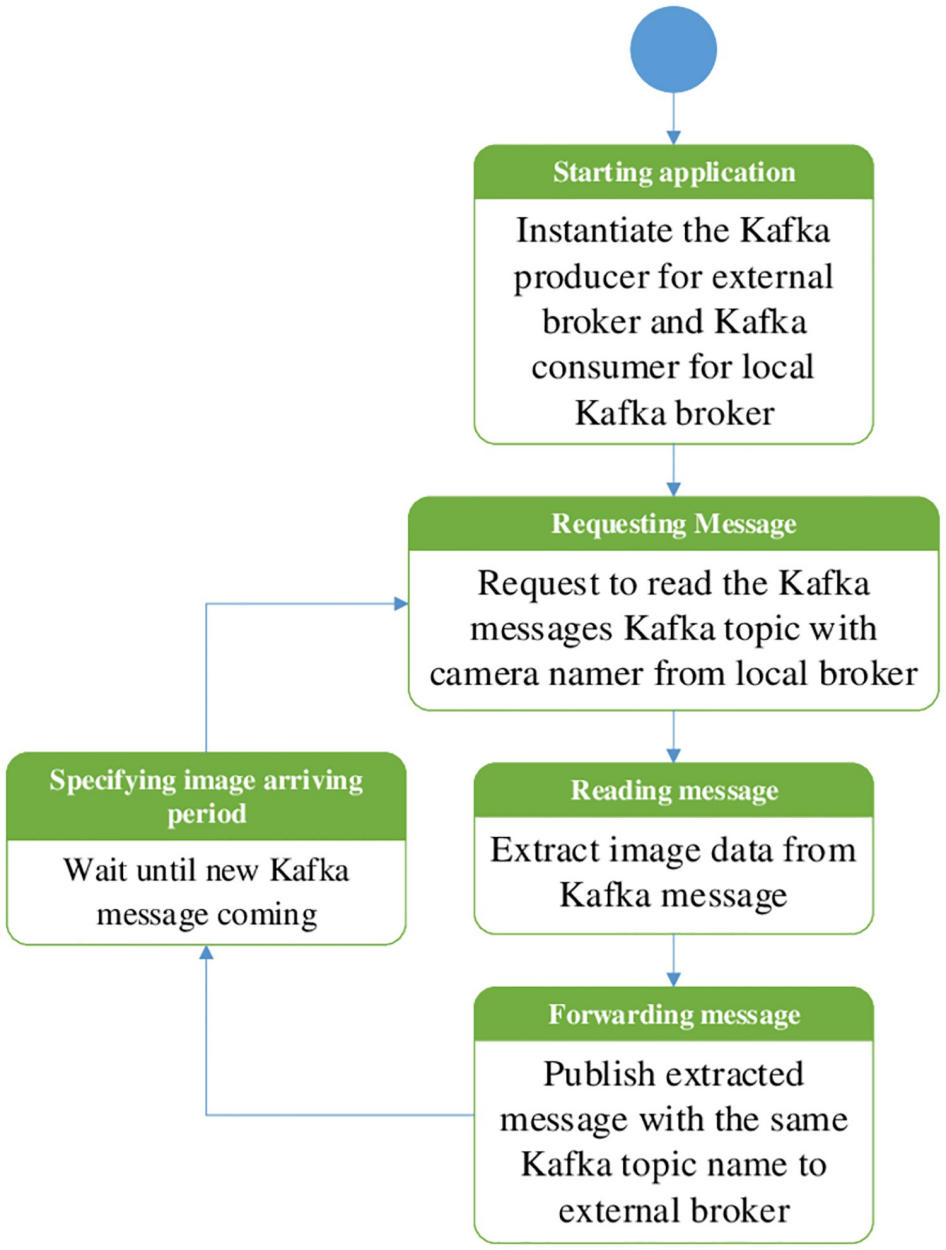
Operational flow chart of traffic controller box application to relay locally received messages to external Kafka broker.

The third function of our application is forwarding the locally received messages to the external broker, and the operational flow chart is shown in Fig 8. This program instantiates another Kafka consumer for the local broker and another Kafka producer for the external broker. The last incoming image message from the local broker is read and published to the external broker with the same Kafka topic name.

**Fig 9:**
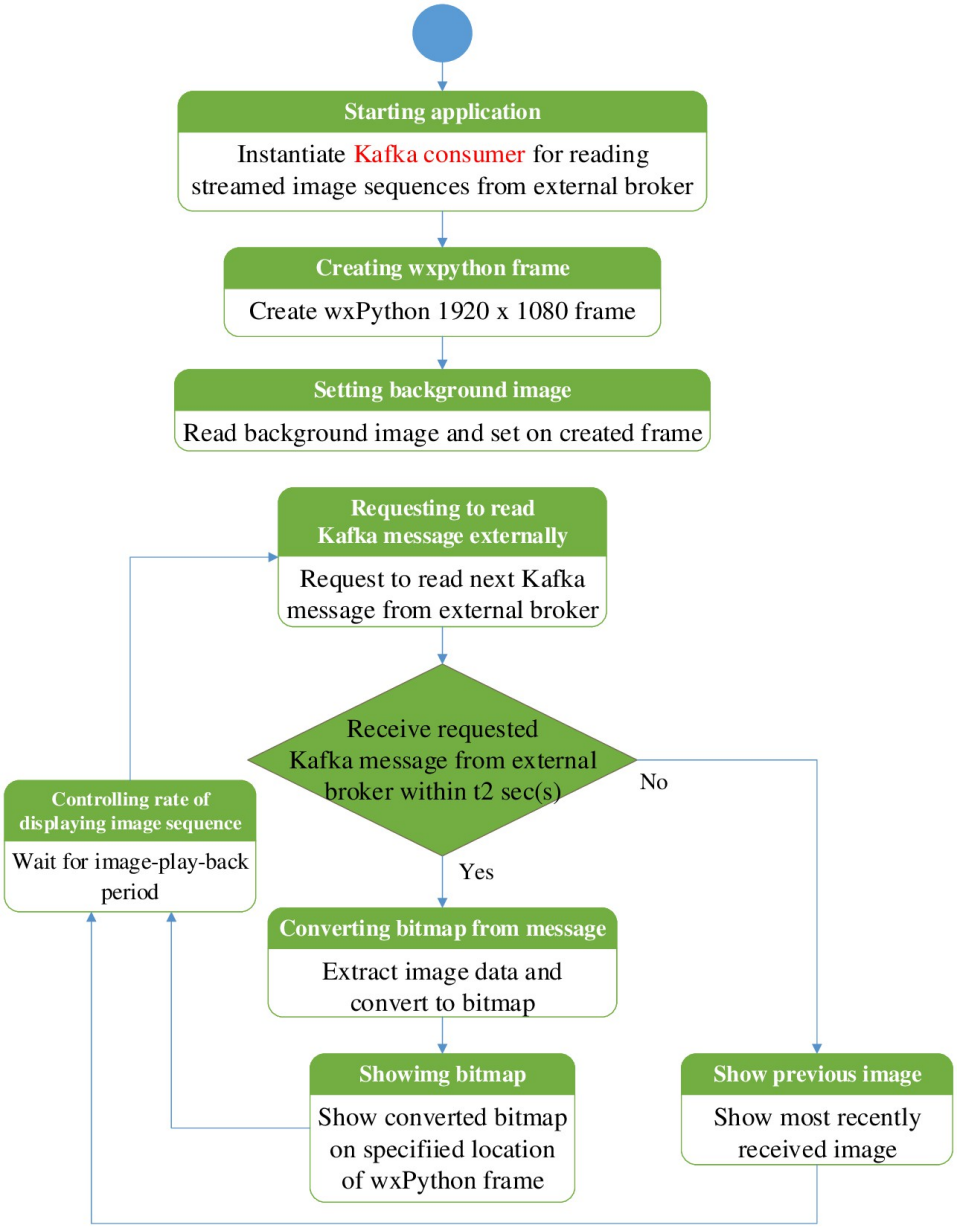
Operational flow chart of external Kafka consumer application in central traffic command centre.

Another Intel^®^ NUC is used as the external cloud facility considered as the central traffic police command center. An external broker serves two functions. The first one is to serve as an external Kafka broker that receives the incoming Kafka messages from local brokers. Another function is to serve as the Kafka consumer by reading and displaying image sequences from the Kafka messages from the external broker, and the flow chart of function is shown in Fig 9. The function here for the monitoring application is operable, similarly to the monitoring application of the local brokers. The only difference is that all the messages are read from the external Kafka broker.

For the storage system, Kafka brokers at both local and external brokers store the incoming Kafka messages as a log file for each mesh node. This storing is needed when traffic police users may need to re-watch the previously recorded image sequences. Those further analyses can be done easily in Kafka by reading the log files stored in the external broker of the traffic data cloud.

Dell laptop latitude E6400 is used as the Ryu controller. At the Ryu controller, the re-routing application is running as the northbound interface application. This application checks the mesh nodes’ reachability status. If an unreachable case occurs, the re-routing scenario occurs to re-route the data plane and control plane traffic from the mesh nodes to the default local brokers.

## Outdoor testing

### Setting of actual testbed component installation on target Phaya Thai Road

In this Section, the steps of actual implementation for the real outdoor testbed at the target road segment as discussed in Section “Proposed Simple Fault-Tolerant Near Real-Time Wireless Image Sequence Streaming Cloud”. Fig 3 shows the topology of the real outdoor testbed.

**Fig 10:**
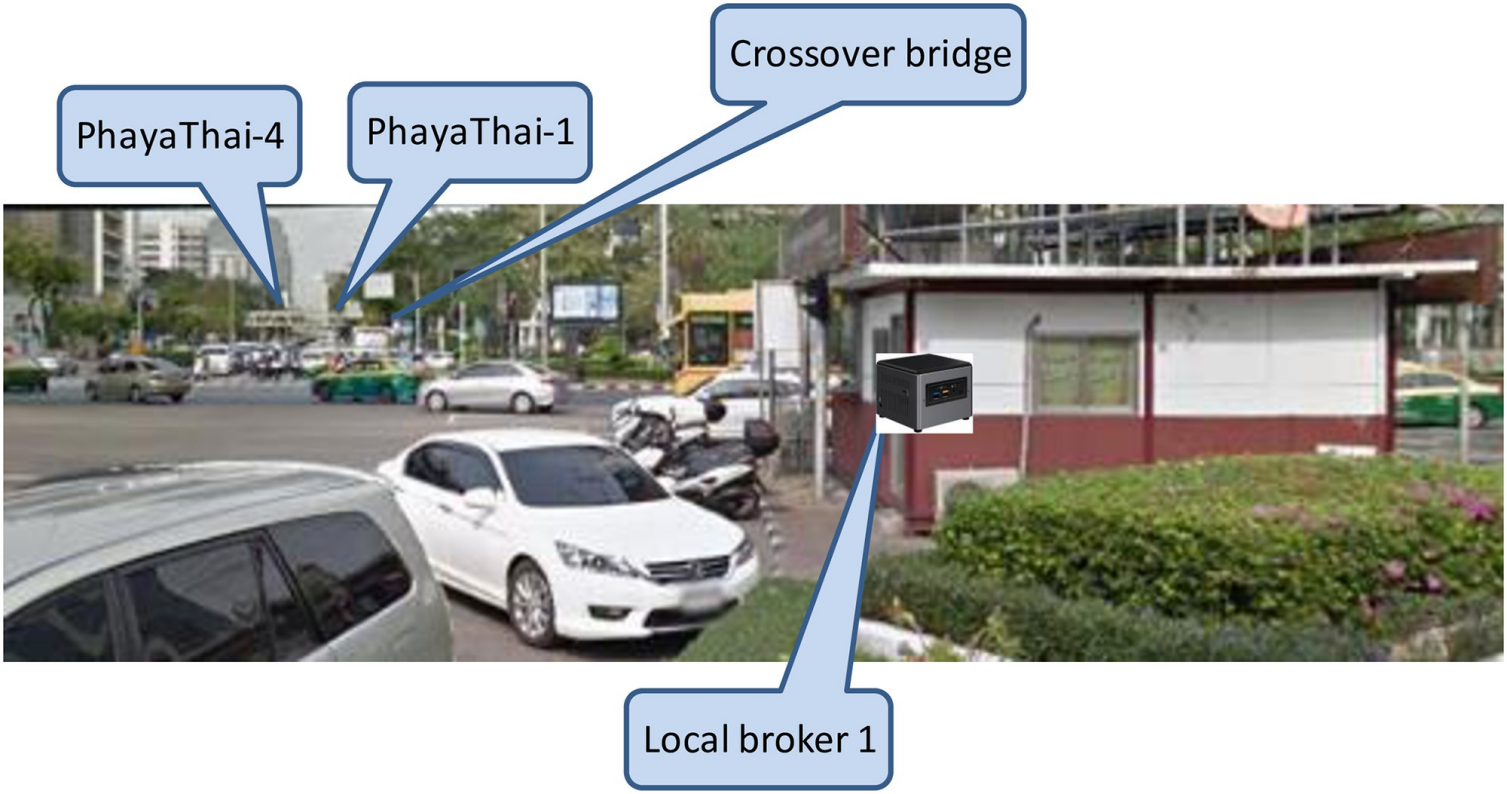
Location of local broker 1 at the junction of Phaya Thai road and Rama 4 road (near Sam Yan).

**Fig 11:**
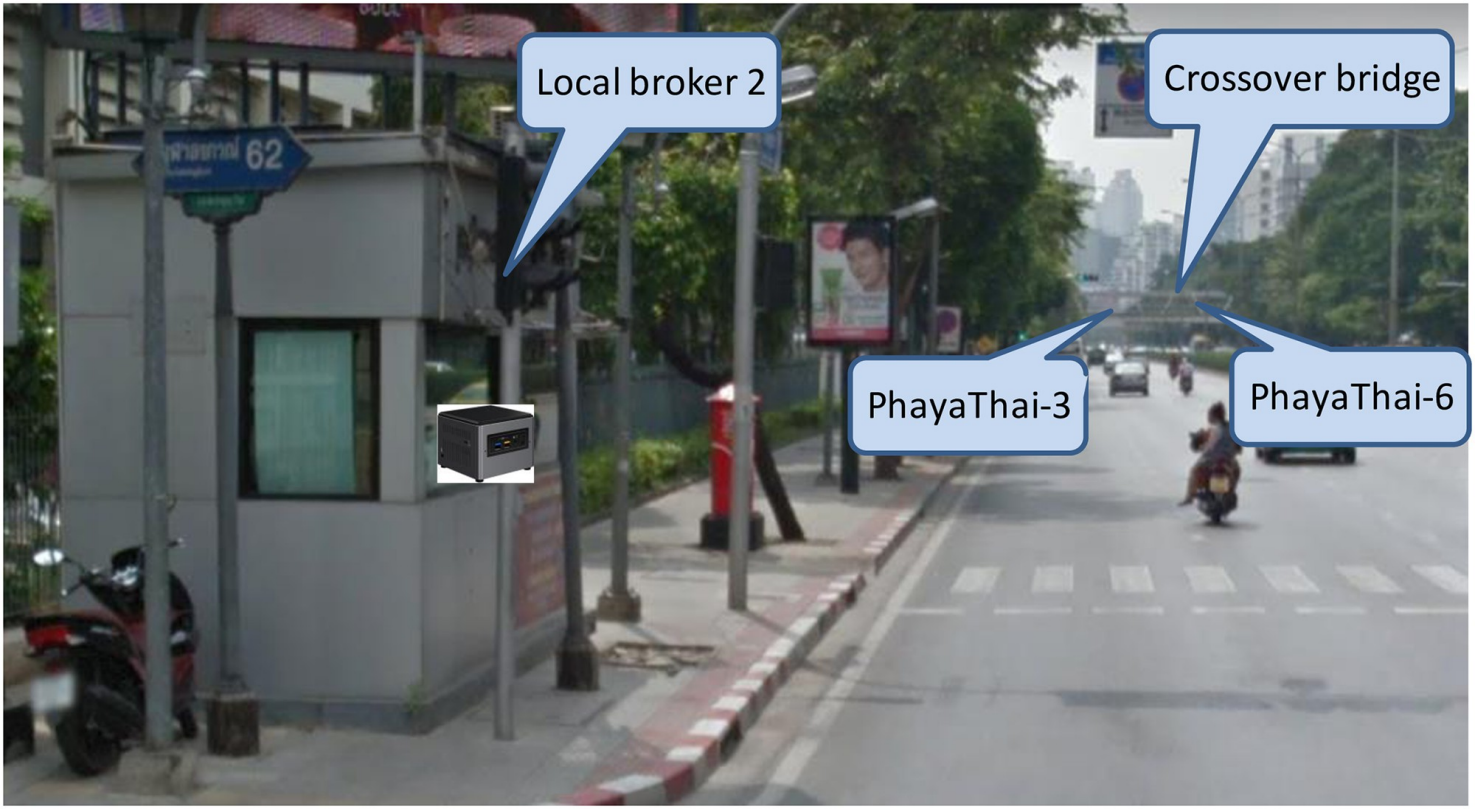
Location of local broker 2 at the junction of Phaya Thai Road and Chulalongkorn Soi 12 (near Mahboonkrong).

**Fig 12:**
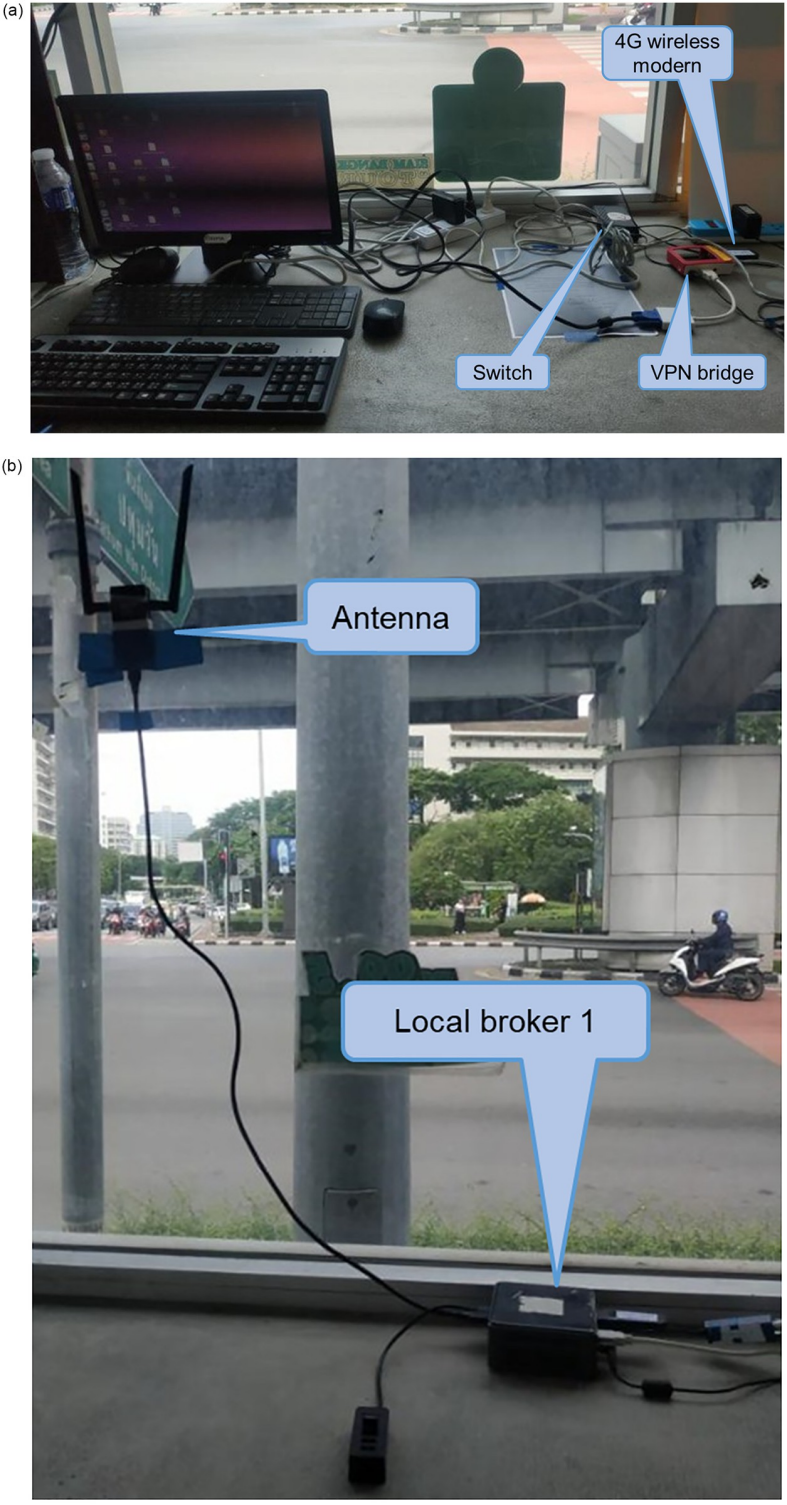
Installation of local broker 1 at specific traffic police controller box in (a) and (b).

**Fig 13:**
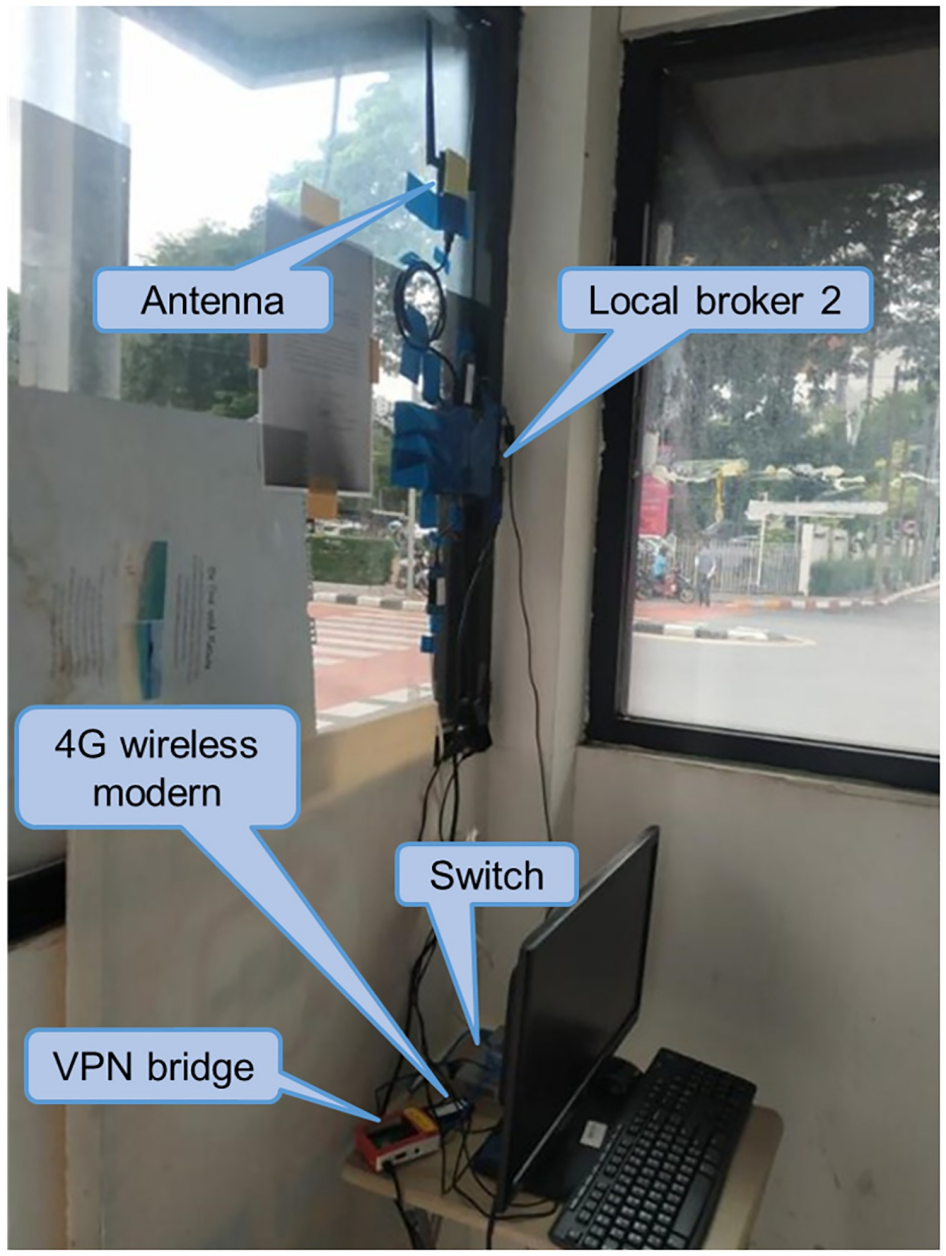
Installation of local broker 2 at specific traffic police controller box.

**Fig 14:**
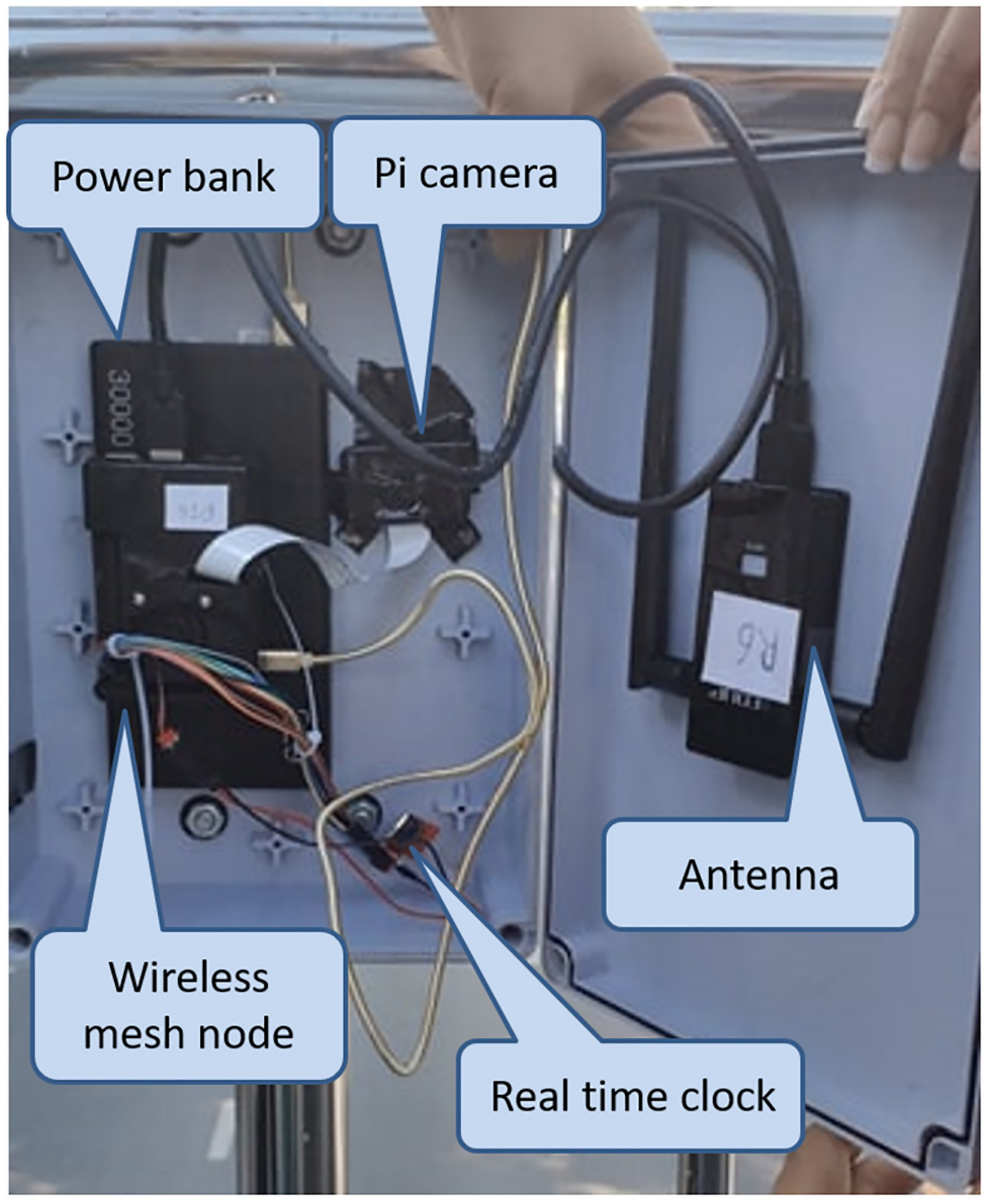
Components for wireless mesh node inside waterproof box.

The locations of the local brokers and external broker are mentioned in Section “Proposed Simple Fault-Tolerant Near Real-Time Wireless Image Sequence Streaming Cloud.” The locations of the local broker 1 and 2 are shown in Figs 10 and 11 respectively. Figs 12 and 13 show the installation of local brokers 1 and 2 inside the traffic police controller boxes respectively. Mesh nodes are installed inside the waterproof boxes as shown in Fig 14. These boxes are attached to the fence of the crossover bridges on Phaya Thai road.

Zookeeper and Kafka broker are installed at the local brokers and external broker as we mention in Section “Proposed Simple Fault-Tolerant Near Real-Time Wireless Image Sequence Streaming Cloud.” In the beginning, the Zookeepers and Kafka brokers are needed to be up and running. A real-time clock (RTC) module ds1307 is attached to each Raspberry Pi to synchronize the clock.

For the underlay SDWMN network, the detail information is mentioned in [20]. Before running the Kafka producer application at the Raspberry Pi’s, the message forwarding application mentioned in Section “Operational Flow Charts of Kafka Producer and Consumer Applications in Simple Fault-Tolerant Near Real-Time Wireless Image Sequence Streaming Cloud” is needed to run first. After setting the forwarding application and the Kafka producer application, the monitoring application mentioned in Section “Operational Flow Charts of Kafka Producer and Consumer Applications in Simple Fault-Tolerant Near Real-Time Wireless Image Sequence Streaming Cloud” is run at each local broker and an external broker. The resolution of each JPEG format image is chosen to be 180p which is 320 x 180 resolution. This 180p resolution is clear enough for the policeman to know the condition of the road.

The image capturing and sending interval time is set to 10 secs, and the maximum threshold of waiting time is set to 15 secs (image capturing and sending interval time + extra 5 secs). The image capturing and sending interval time and the maximum waiting time threshold is large because of the obstacles, which can block the wireless link.

Kafka producer applications at PhayaThai-1, 3, 4, and 6 produce images to the default local broker only because the local broker switching mechanism at these mesh nodes can have a large delay since these nodes are 3-hop away from other local brokers. Kafka producer application with local broker switching mechanism is run at PhayaThai-2 and 5.

**Fig 15:**
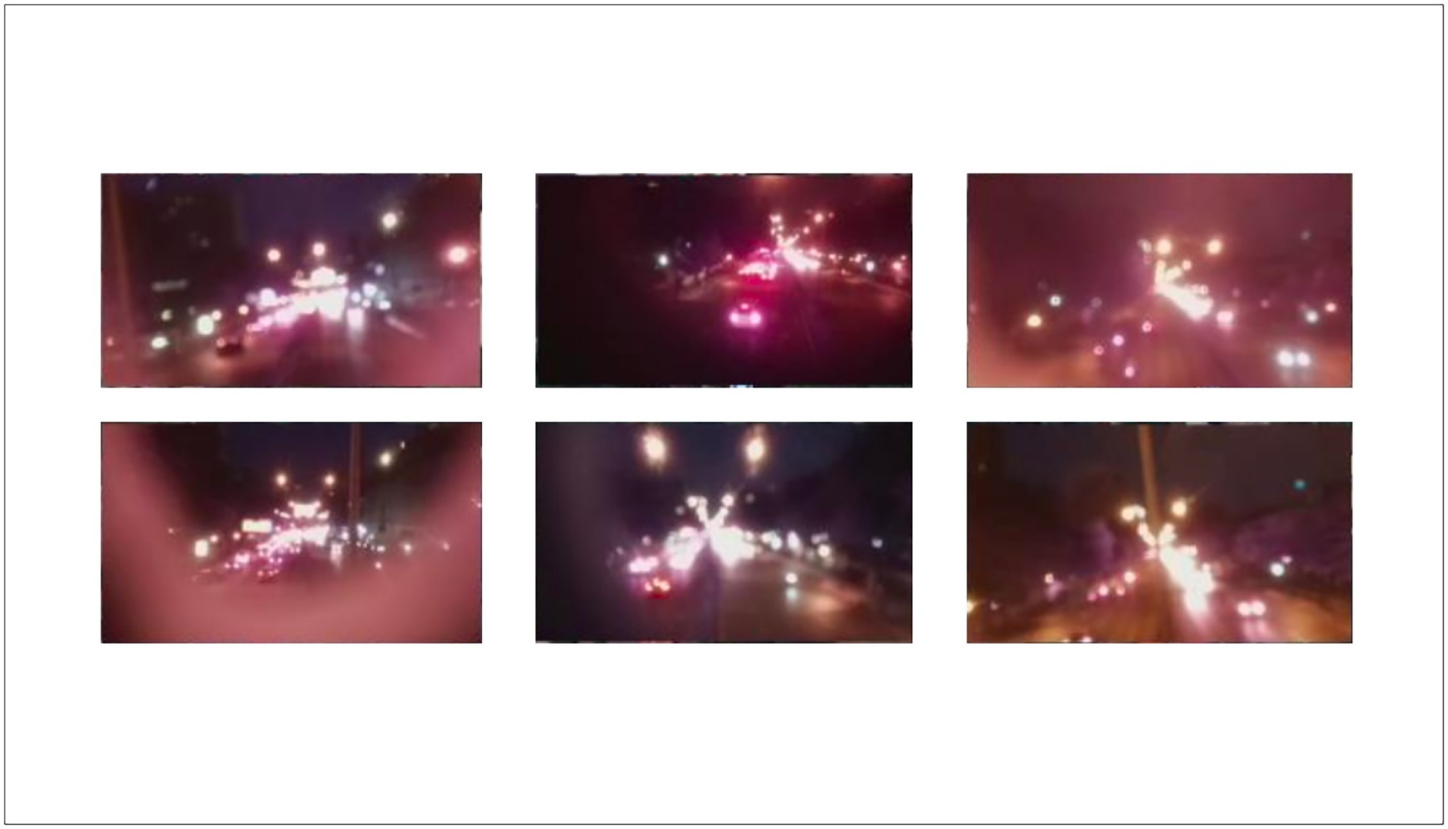
Snapshot example of monitoring application at external broker.

The testing has started from 5:40 P.M. 17^th^ November 2020 (Tuesday) to the next day at 6:40 A.M. 18^th^ November 2020 (Wednesday). Since the testing has started around the rush hours of road traffics, there is traffic congestion during the starting period. This congestion can affect the wireless link between the mesh node and local brokers. The big vehicles, e.g. bus, trucks, can temporarily block the line-of-sight of wireless links between the local brokers and mesh nodes. The snapshot examples of monitoring application is shown in Fig 15.

### Measurement result of end-to-end average latency and image loss percentage

To measure the end-to-end average latency, the RTC time of all Raspberry Pi’s are synchronized before the streaming application starts. The latency is calculated with the subtraction of mesh node sending time from image message reading time at the monitoring application of each local broker and an external broker. To measure the image loss, an additional frame number counter is added to the Kafka message. Therefore, the image loss can be measured at the monitoring application by looking up the frame number inside the Kafka message. The end-to-end average latency and image loss percentage have been collected for 13 hours of the testing result of the outdoor testbed.

**Fig 16:**
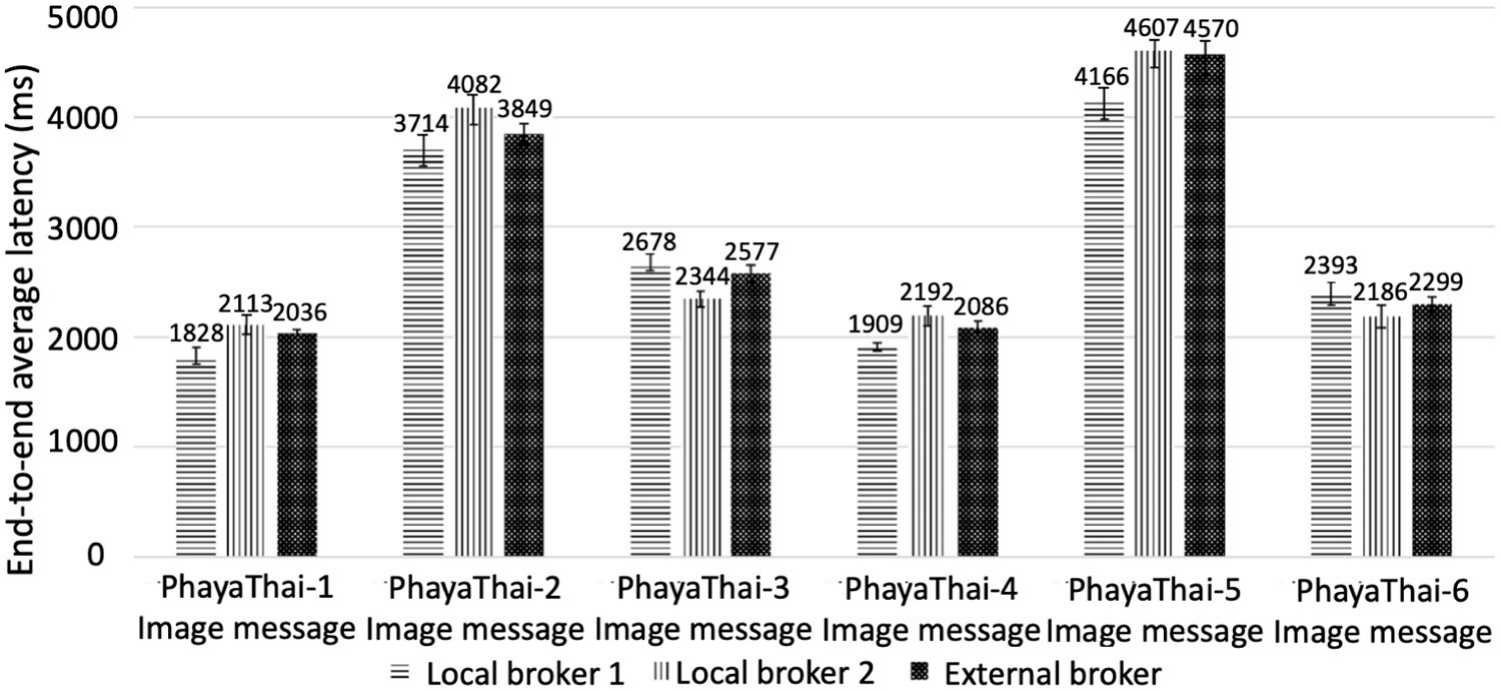
End-to-end average latency of image messages received at each broker for each wireless node (Phaya Thai road test).

**Fig 17:**
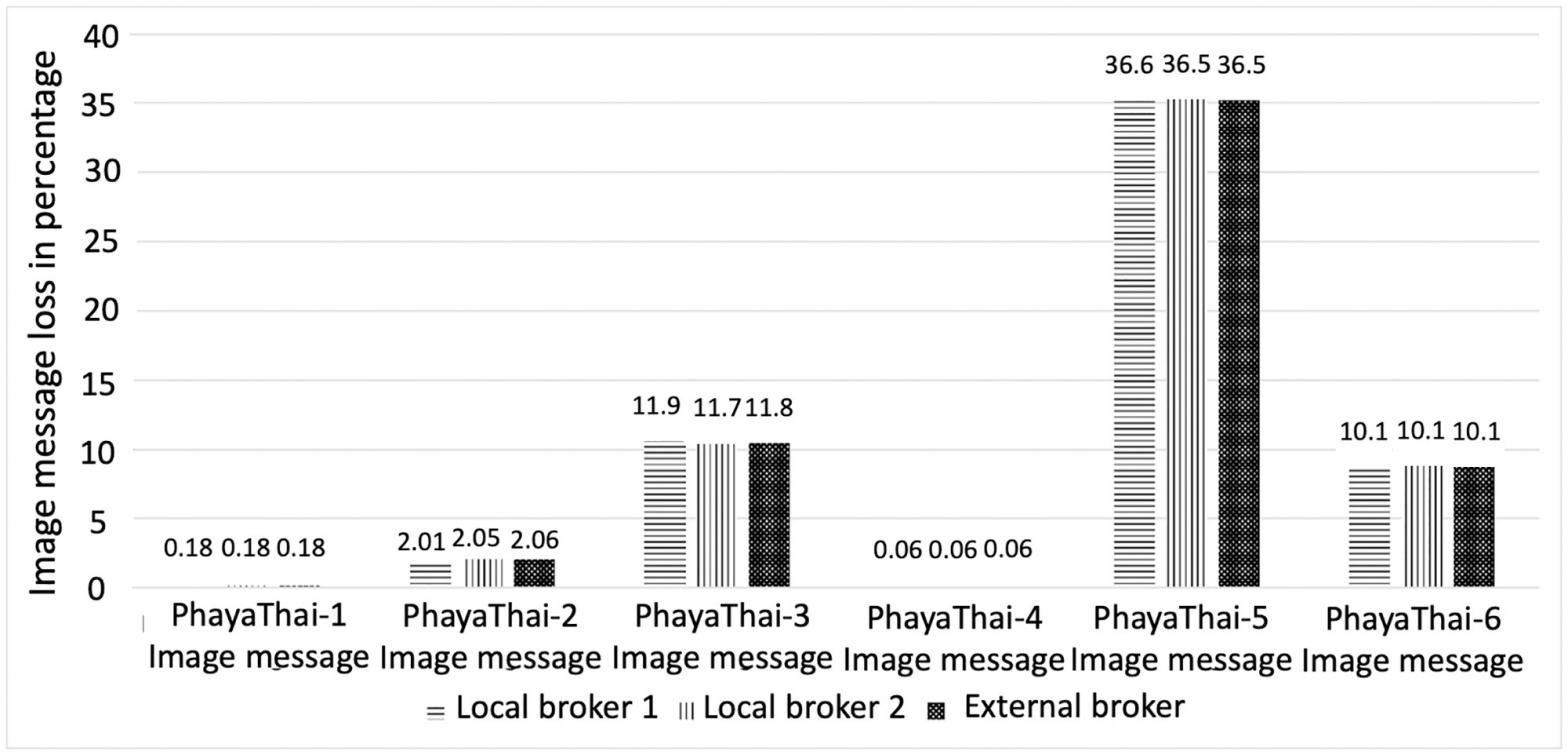
Image message loss percentage measurement by traffic monitoring application at local brokers and external broker (Phaya Thai road test).

The result of the graph of end-to-end average latency with 95% confidence interval and the image loss measurement by traffic monitoring application at local brokers and external broker are shown in Figs 16 and 17 respectively. One-hop distance wireless nodes to local brokers give a smaller delay compared to the two-hop distance nodes. It is because increasing the hop results in the increasing delay. Therefore, the middle node (PhayaThai-2 and 5) has a larger delay time than the one-hop node (PhayaThai-1, 3, 4 and 6). For the PhayaThai-1 and 4, there is the obstacle such as some big car between the mesh nodes and the local broker 1. Because of the local broker 1 location, the line-of-sight wireless link can be blocked when congestion occurs in front of the traffic police controller box. However, this is only is temporarily only, and therefore, the image loss percentages of these two nodes are small (less than 1%) compared to other mesh nodes. For PhayaThai-3 and 6, due to the location of the local broker 2, there are much more obstacles near local broker 2. The traffic police controller box for the local broker 2 is located on the pavement as shown in Fig 11. Trees grow along the pavement, and tree leaves can block the wireless link between local broker 2 and PhayaThai-3 and 6. Line-of-sight wireless links can also be temporarily blocked when congestion occurs near the traffic police controller box. Therefore, the image loss percentage of these two nodes is large compared to other one-hop distance mesh nodes (PhayaThai-1 and 4).

**Fig 18:**
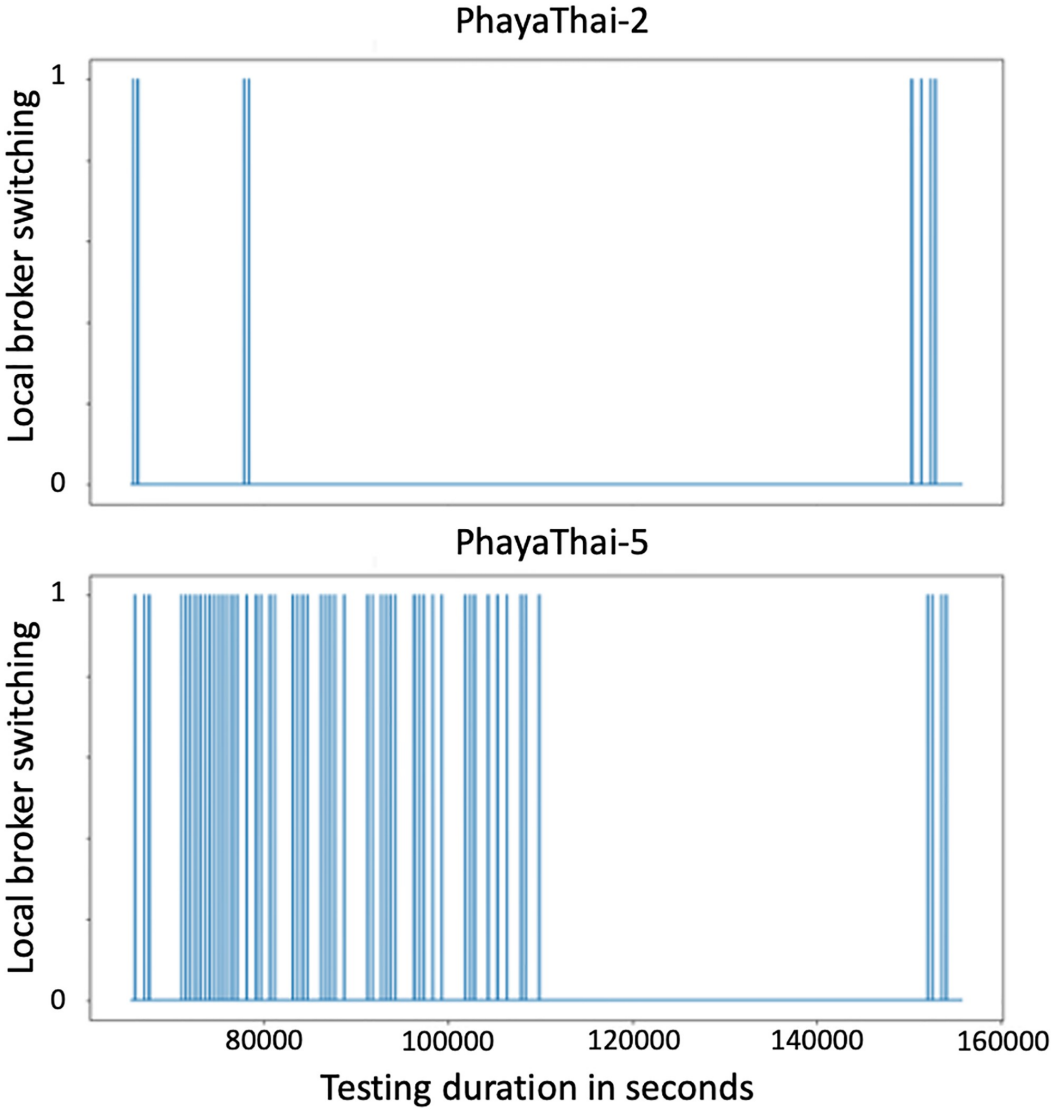
Local broker switching time of PhayaThai-2 and PhayaThai-5 in seconds (Phaya Thai road test).

**Fig 19:**
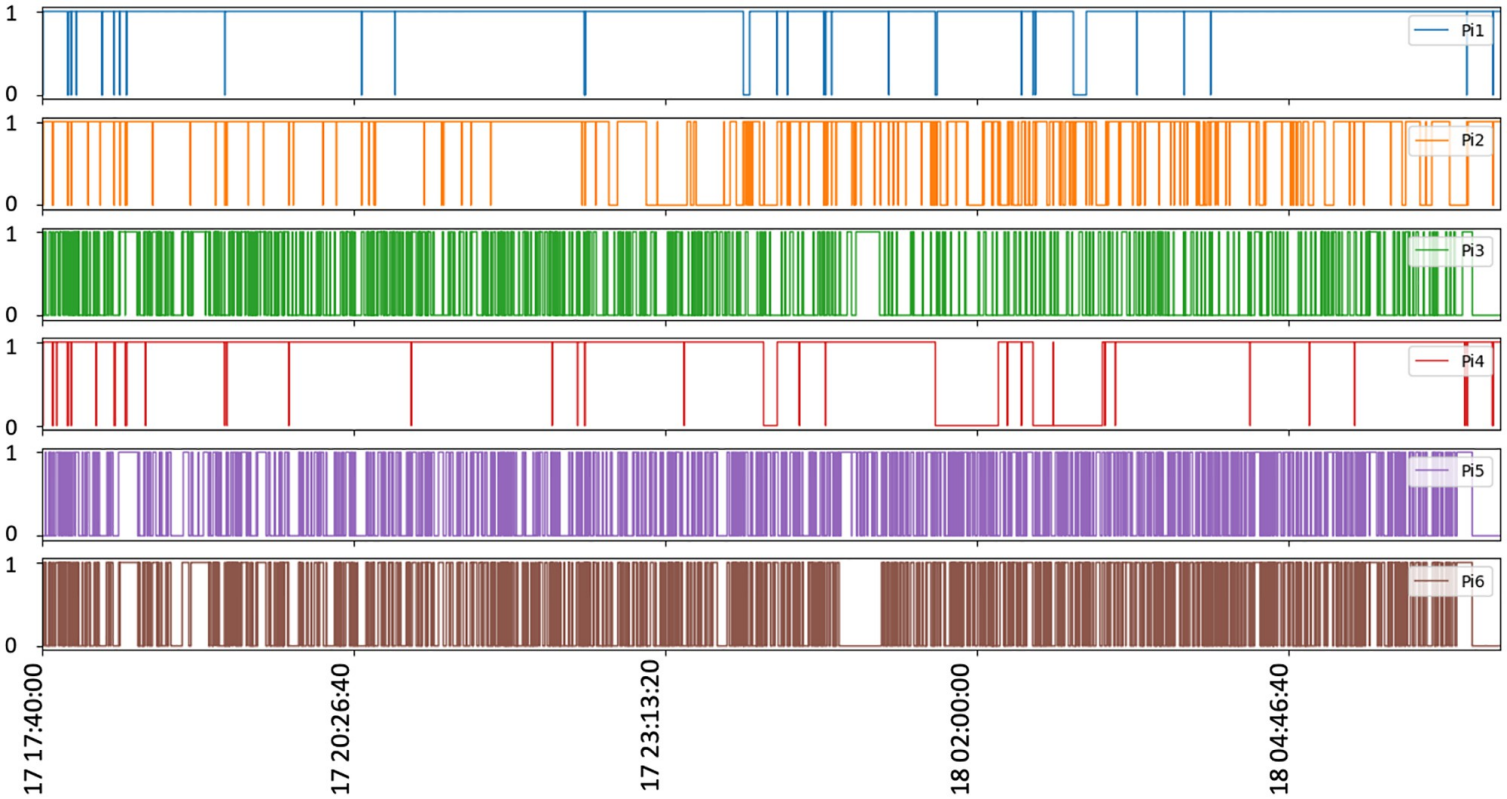
Practical operation status of SDWMN control plane for 13 hours testing (Phaya Thai road test).

Fig 18 shows the local broker switching time. Here, the x-axis means testing period in seconds, and one at the y-axis means the mesh node switches the local broker and sends image messages. 0 at the y axis means that the mesh node sends image messages to the current_used_broker. Fig 18 shows that PhayaThai-5 node switches the local broker too frequently during the beginning of the testing period. This is because of the line-of-sight blockage between the local broker 2 and PhayaThai-6 node. As a result, the image loss percentage is increased at PhayaThai-5 node. However, the end-to-end average latency is kept around desired limit which is less than 5 seconds. According to the traffic police suggestion, 5-second late information of the road traffic condition can be acceptable to control the traffic signal light. End-to-end average latency is kept around this amount because of the local broker switching scenario. When the current_used_broker is not reachable, the Kafka producer application at the PhayaThai-5 node will switch to the local broker of the other side. This is in addition to the re-routing algorithm of the SDWMN underlay network. Having only the re-routing algorithm provided by the underlay SDWMN network will keep trying to get to the same destination. For PhayaThai-5 case, the reachability from the local broker 2 is unreliable. As shown in Fig 19, the reachability status of the SDWMN control plane to the PhayaThai-3, 5 and 6 from the local broker 2 is fluctuating a lot. This SDWMN uses the in-band control scenario. The data packet and control packet are sent with only one interface. The node reachability means that the SDN controller is disconnected from the mesh nodes in the control plane in [20]. During the operation, when mesh nodes are disconnected from the SDN controller, Fig 19 shows 0 at each graph. When the node reconnects to the SDN controller, Fig 19 shows one at each graph. The reachability is measured via the default gateway in the local broker. Therefore, unreachability of PhayaThai-5 means local broker 2 and PhayaThai-5 are not connectable via the primary route or alternative route. This does not mean the PhayaThai-5 is not reachable to the local broker 1. Therefore, having only one layer restoration cannot maintain the satisfactory service experience. Because of this disadvantage of having only one layer restoration, in this paper, we proposed the two layer restoration framework for network as well as application layer.

In Fig 19, the connection between the SDN controller and PhayaThai-3, 5 and 6 have fluctuated a lot. This is because of the local broker 2 location and the temporary blockage of the line-of-sight wireless link between the local broker two and mesh nodes. Because of the connection between the local broker 2 and PhayaThai-6, PhayaThai-5 connection to the local broker 2 is also not good, as we can see in Fig 18. However, this Fig 18 shows only the control plane reachability. The data plane traffic can go to an alternative local broker e.g., PhayaThai-5 can successfully switch the destination, local broker, to an alternative local broker. Because of this switching capability of the local broker, end-to-end average latency and the image loss percentage can be reduced. However, such losses can be reduced even further in future work by sending the flag to signal the switch back to the local broker instead of sending the whole batch of the image sequence. Another reason for the high image loss percentage is that PhayaThai-5 switches the local broker too often as shown in Fig 18. PhayaThai-5 switches the local broker when the reply message for the last image message from the current_used_broker does not arrive within the maximum waiting time threshold.

Although PhayaThai-5 switches to the alternative local broker (local broker 1), the wireless link between PhayaThai-5 and local broker 1 is not good. Despite switching to the alternative local broker (local broker 1), the maximum waiting time threshold is not long enough. PhayaThai-5 switches to the default local broker (local broker 2). Therefore, in Fig 18, the local broker switching time for PhayaThai-5 has occurred twice most of the time, which means Kafka producer for PhayaThai-5 is switching the local brokers back and forth after reaching the max_images_in_batch by the current_used_broker. Because of local brokers switch too often, the image loss percentage has increased. It is noticeable from Fig 17 for the outdoor testing that the image loss percentage can be huge for PhayaThai-5 with the two-hop route to reach its destined local broker. Such high image losses would indeed be considered not negligible when applied in reality. And that is why the system architecture proposed in this paper also tries to include redundancies. Here, we have designed the camera angles to overlap for the nodes installed on the same crossover bridges, for instance, for PhayaThai-2 and PhayaThai-5 nodes. So, when an active path from one node is disrupted by recurrent line-of-sight obstacles like big-bus passing, that node’s intended camera view angle is erroneous. However, should the other node with its installed position at the opposite roadside still function correctly, the traffic police could still see the overall road traffic congestion in that area. It should also be noted that despite such inherently included equipment redundancy, the cost of engineering the proposed system remains far lower than the cost of building the currently used wire-based CCTV system in the city of Bangkok. Hence, if one could demonstrate the feasibility of using this wireless ad-hoc basis to design our near real-time road traffic monitoring application, we believe the practical deployment should be justified easily.

Switching the local broker too often can result in high image loss because both local brokers cannot receive the entire image sequence. This result thus depicts the applicability range boundary of the proposed system. That is, the local broker switching can cause the image loss because a batch of the image sequence is transmitted to the default local broker to check whether the default local broker is reachable from the PhayaThai node or not. This checking results in high image loss during the network traffic congestion condition. Another boundary is that during the increased network traffic congestion or low bandwidth condition, the image sending node is trying to stream the images by sending the image sequence to the local brokers back and forth. During that time, the image sequences from the node are lost and causing a high image loss percentage.

At the external broker, the image message loss percentage is similar to at the local brokers because the local brokers forward image messages received at the local brokers. Therefore, the additional image message loss has occurred because of the Internet connection only. However, the image loss percentage is under the acceptable condition at both local brokers and external broker.

In this testbed, the testing period is from 5:40 P.M. 17^th^ November 2020 to 6:40 A.M. 18^th^ November 2020 which is night time. During the night time, the traffic congestion is less than the daytime road traffic congestion. However, the beginning of the testing period is the most congested period of the whole day which is from 6 P.M. to 9 P.M. During that period, the system performance of the left side network (local broker 1, PhayaThai-1, 2 and 4) is under the acceptable condition. For the right side of the network (local broker 2, PhayaThai-3, 5 and 6) the system performance of PhayaThai-3 and 6 is under the acceptable condition except the PhayaThai-5 node whose image loss percentage is higher than the other nodes. However, because of the local broker switching scenario, the end-to-end average latency is under the acceptable condition. Apart from that switching scenario, the cameras are pointed to the similar road condition, the loss of information from one camera is acceptable for the traffic police to know the road condition. Since the tested period has already included the most congested period (i.e. during evening rush hours) of the whole day, the daytime testing result value should be similar to or better than the result of this testing period.

There are limitations to the proposed framework. One of the limitations of the proposed framework is that the restoration of network layer and application layer are not cooperated with each other. This might lead to the unnecessary restoration occurrences. Another limitation is that the whole batch of image sequence is sent and checked for the reachability at the application layer by only the last message of the batch. This might lead to a Kafka producer trying to send its generated images an unreachable local broker. All these limitations could potentially give the high image message loss percentage. However, in practical operations of traffic monitoring, the traffic police on-duty would not be always attentive to the traffic monitoring dashboard. So, losing some images would merely cause the latest updated images to be frozen on the monitoring screen. And especially when the traffic is congested, information would not be lost much because an image of stopping vehicles in queue would remain relatively unchanged. And once the new images of the same location have been later on retrieved, the traffic police would be able to get the new information eventually. So, performance in terms of image losses can still be rationally traded-off with the improved cost effectiveness of the proposed system.

## Comparison between OLSR and proposed system

**Fig 20:**
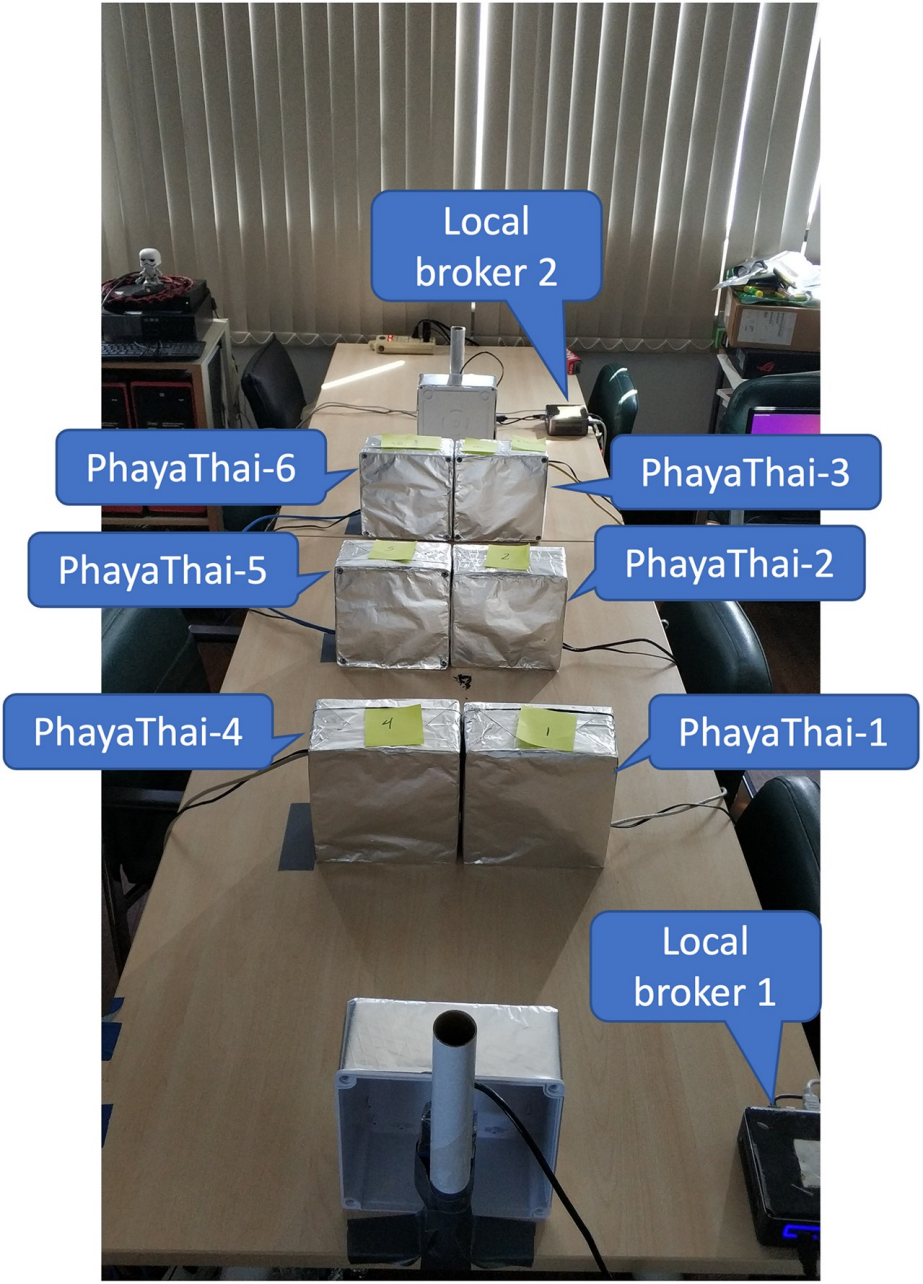
Laboratory-based emulated outdoor network.

In this Section, the proposed system with the SDWMN network is compared with a well-known wireless ad-hoc network protocol, OLSR [23]. To be fair, the comparison is made under the controllable emulated network built at the laboratory. The emulated network has a physical topology that has been recalibrated to match topologically with that of the outdoor testbed. For the outdoor testbed, the longest per-hop range of the wireless mesh nodes is 200 to 300 meters apart along the road length at the location of crossover bridges where the nodes can be installed. However, at the laboratory-based emulated network, the physical inter-spacing range of the wireless mesh nodes must be shrunk to the workbench dimension inside the laboratory. Therefore, the antenna gain effect must be reduced to have a miniaturized coverage area. The physical installation of the emulated testbed is shown in Fig 20. In the emulated testbed, the wireless mesh nodes are 50 centimeters apart along the table length instead of 200 to 300 meters along the road length. Aluminum foil has been used to wrap in required multiple layers around the plastic box in which the wireless mesh nodes are installed to reduce the antenna gain effect. The wireless nodes are placed carefully to have the desired throughput and coverage area that match the real outdoor SDWMN network testbed topology.

### Parameter setting

SDWMN underlay network is compared with OLSR in terms of the restoration time when one node is down. The middle nodes must be re-routed to the desired destination nodes, e.g., PhayaThai-2 and 5, to the local broker 1 and 2, respectively, when the primary route fails. When the primary route is available, the route should be restored to the primary route. Therefore, in this comparison, the time needed for re-routing to the alternative route and the time needed for restoration to the primary route is compared using the OLSR and SDWMN network. In this comparison, the left side of the wireless mesh nodes has been used because the network topological configuration is the same for the left side (local broker 1, PhayaThai-1, 2 and 4) and for the right side (local broker 2, PhayaThai-3, 5 and 6).

**Fig 21:**
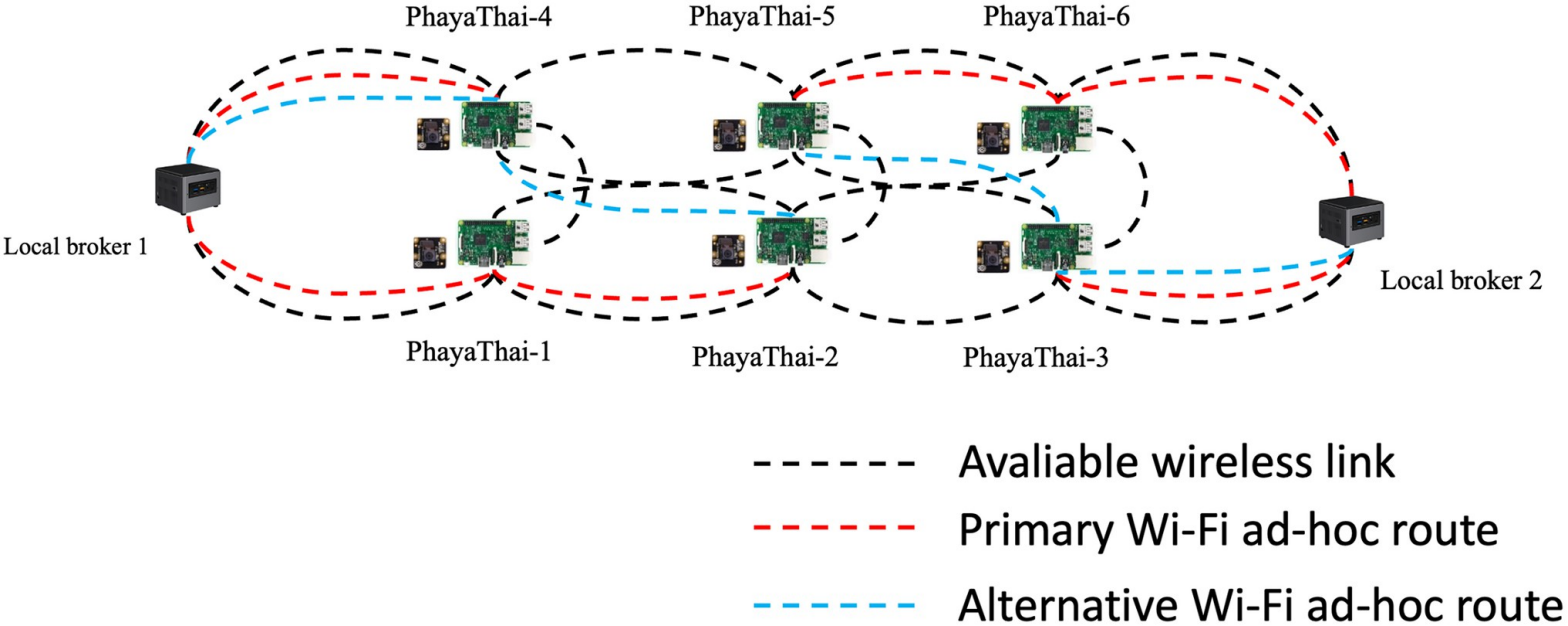
Routing of OLSR network.

**Fig 22:**
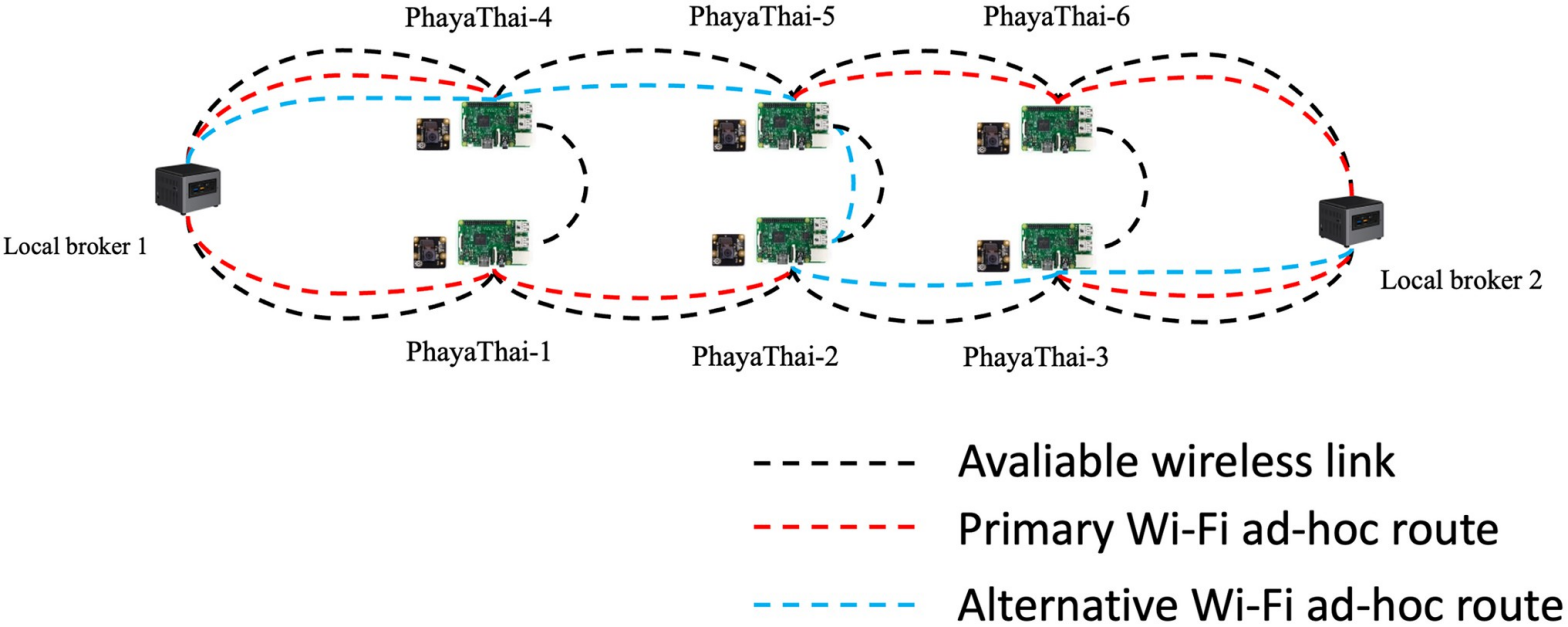
Routing of SDWMN network.

In the experiment, the interface of PhayaThai-1 node is up and down for a certain amount of time (here 3 minutes) to trigger the re-routing event to the alternative route and restoration event to the primary route during the testing period. The primary route and alternative route for the OLSR network and SDWMN network are shown in Figs 21 and 22. The throughput of PhayaThai-4 node is limited to have controllably different end-to-end capacity for the primary route and alternative route. With the OLSR protocol, the route is determined by the link state. By having the throughput limitation at PhayaThai-4, the routing for PhayaThai-2 will be under control. Therefore, the primary route for PhayaThai-2 to local broker 1 with the OLSR underlay network is PhayaThai-2, 1 and local broker 1 while the alternative route for the same destination as the primary route is PhayaThai-2, 4 and local broker 1. For the SDWMN underlay network, the primary route for PhayaThai-2 to local broker 1 is the same as the OLSR underlay network. However, the alternative route for PhayaThai-2 to the same destination as the primary route is PhayaThai-2, 5, 4, and local broker 1. The reason to use this as an alternative route for PhayaThai-2 with SDWMN underlay network is to achieve the same network routing mechanism as the outdoor testbed.

For the OLSR protocol, discovering the network and link state is done by broadcasting HELLO messages to neighboring mesh nodes. With the HELLO message, the information of neighboring nodes is exchanged, and the route is set up for the data plane. The default interval time for broadcasting HELLO messages is 6 seconds. The control message sending interval time from the SDN controller at SDWMN underlay network is thus set to be the same as HELLO message interval time, which is 6 seconds. To have the faster data rate than the outdoor testbed, the resolution of the image is reduced to 144p resolution. Image resolution is reduced by around half compared to 180p resolution, and the image interval time can be thus proportionally reduced to 6 seconds, where the data rate remains similar to the data rate during the outdoor testbed. To reduce the high end-to-end latency, the buffer memory size of the Kafka producer is set to 1 MBytes instead of 32 MBytes that is the default value. With the reduced buffer size, the image messages unsent during the re-routing and restoration time are cleared. The latency value is more important than the image message loss percentage to have the near real-time image sequence streaming application. This is because messages arriving too late become useless for real-time traffic flow management operations by traffic police.

To measure the re-routing time and restoration time, a one-byte ICMP packet sent to local broker 1 every 0.1 seconds. By looking at the MAC address of the ICMP packets, the current route during that time period can be traced. If the MAC address of the ICMP packet is the PhayaThai-1 address, the current route is the primary route, and if the MAC address of the ICMP packet is the PhayaThai-4 address, the current route is the alternative route for OLSR underlay network. For the SDWMN case, If the MAC address of the ICMP packet is the PhayaThai-1 address, the current route is the primary route, and if the MAC address of the ICMP packet is the PhayaThai-5 address, the current route is the alternative route for SDWMN underlay network. Since this testing aims to compare the re-routing and restoration time of the OLSR underlay network and SDWMN underlay network, the local broker switching scenario is disabled to have a clear comparison, but all the other operations of Kafka producers and consumers are kept exactly the same as in the real outdoor testbed.

Three different comparison cases have been carried out, and the detail of the cases have been mentioned in Table 2.

**Table 2:** Different cases tested for the comparison.

Case 1	Case 2	Case 3
No throughput limitation at PhayaThai-1, 2 and local broker 1	No throughput limitation at PhayaThai-1, 2 and local broker 1	No throughput limitation at PhayaThai-1, 2 and local broker 1
Limit maximum throughput of PhayaThai-4 to be 100kbps during the testing	Limit maximum throughput of PhayaThai-4 to be 50kbps during the testing	Limit maximum throughput of PhayaThai-4 to be 50kbps during the testing
PhayaThai-1 interface is up for first 3 minutes and after that down for 3 minutes and recovered and up for 3 minutes	PhayaThai-1 interface is up for first 3 minutes and after that down for 3 minutes and recovered and up for 3 minutes	PhayaThai-1 interface is up for first 3 minutes and after that down for 3 minutes and recovered and up for 3 minutes when PhayaThai-1 interface is recovered, PhayaThai-4 interface is down
When PhayaThai-1 interface is down, PhayaThai-2 rerouted with alternative route	When PhayaThai-1 interface is down, PhayaThai-2 rerouted with alternative route	When PhayaThai-1 interface is down, PhayaThai-2 rerouted with alternative route
When PhayaThai-1 interface is recovered, PhayaThai-2 restored the primary route	When PhayaThai-1 interface is recovered, PhayaThai-2 restored the primary route	When PhayaThai-1 interface is recovered, PhayaThai-2 restored the primary route

### Result and discussion

Each case mentioned in Table 2 has been carried out for five times for both the OLSR underlay network and SDWMN underlay network. Re-routing time here means that the time needed to change the route to the alternative route when the PhayaThai-1 interface is down. Therefore, the time has been measured after 3 minutes of initial normal running time without any failures incurred. Restoration time here means that the time needed to change the route to the primary route after PhayaThai-1 interface is recovered. Therefore, the time has been measured after 6 minutes of running time. End-to-end latency has been also measured during the experiment. End-to-end latency of particular interest in the experiment here is from PhayaThai-2 to the local broker 1. End-to-end average latency is reported for each primary route and alternative route of OLSR and SDWMN underlay network. The end-to-end average latency is reported then in separated time intervals respectively after re-routing and restoration.

**Fig 23:**
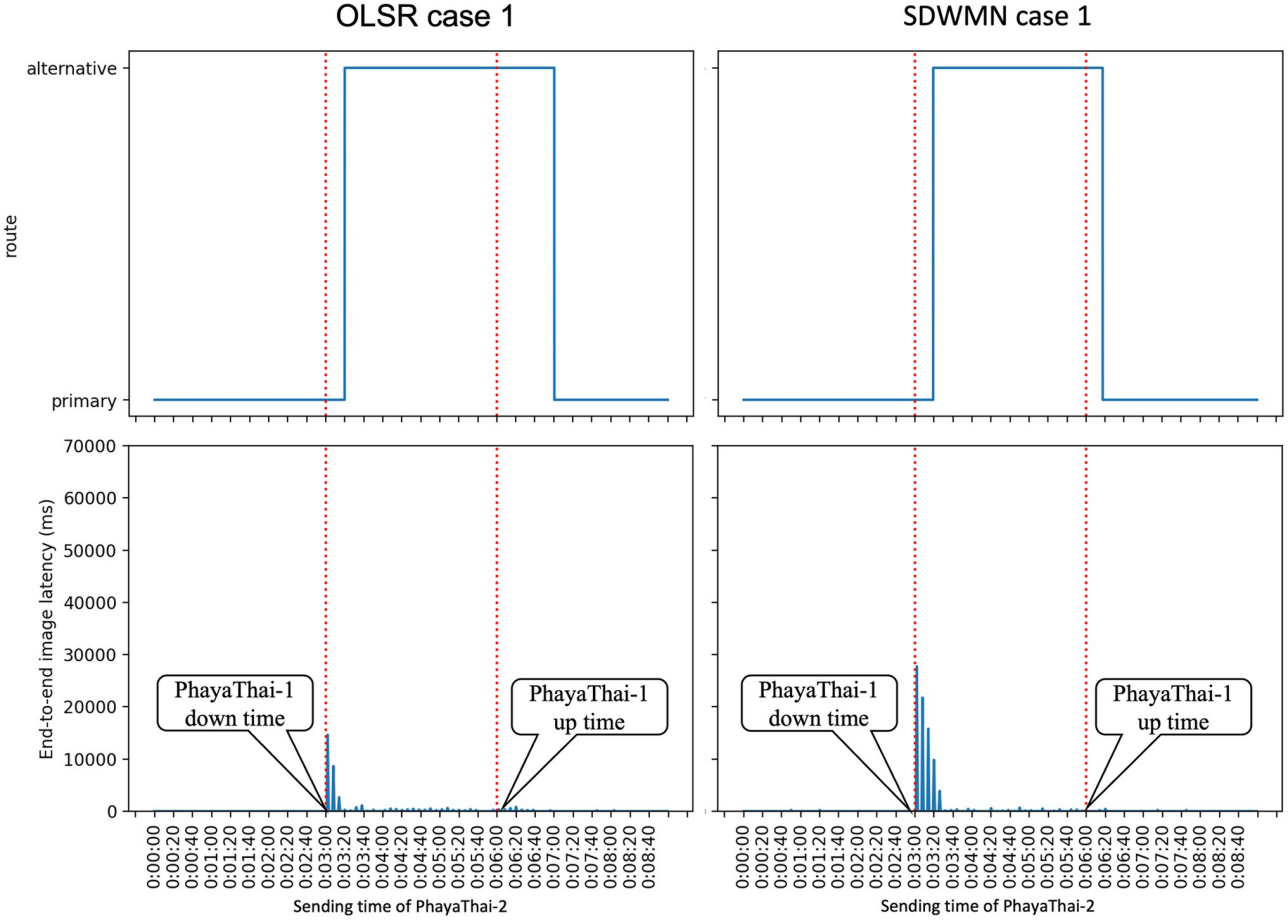
Example of case 1 for OLSR and SDWMN.

**Fig 24:**
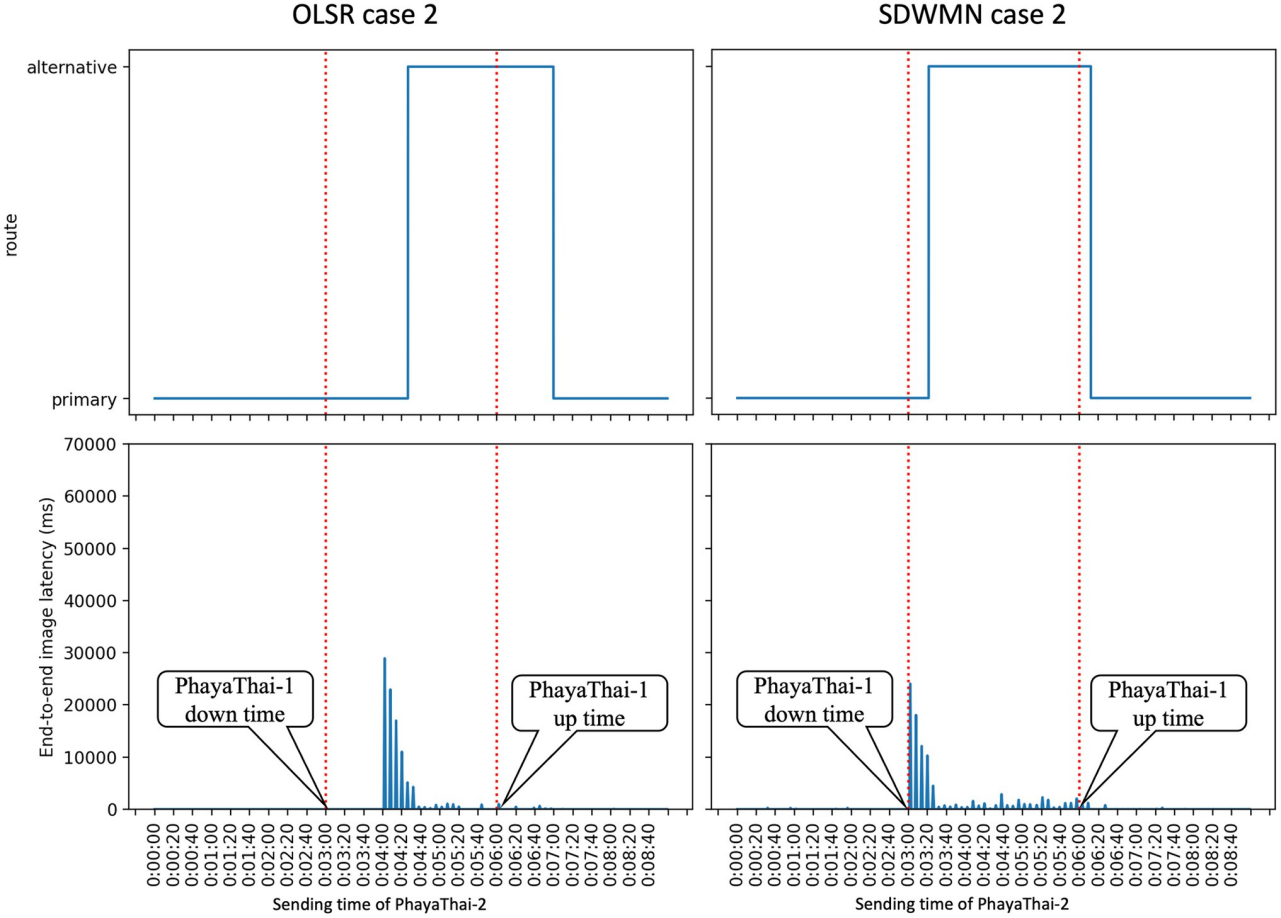
Example of case 2 for OLSR and SDWMN.

**Fig 25:**
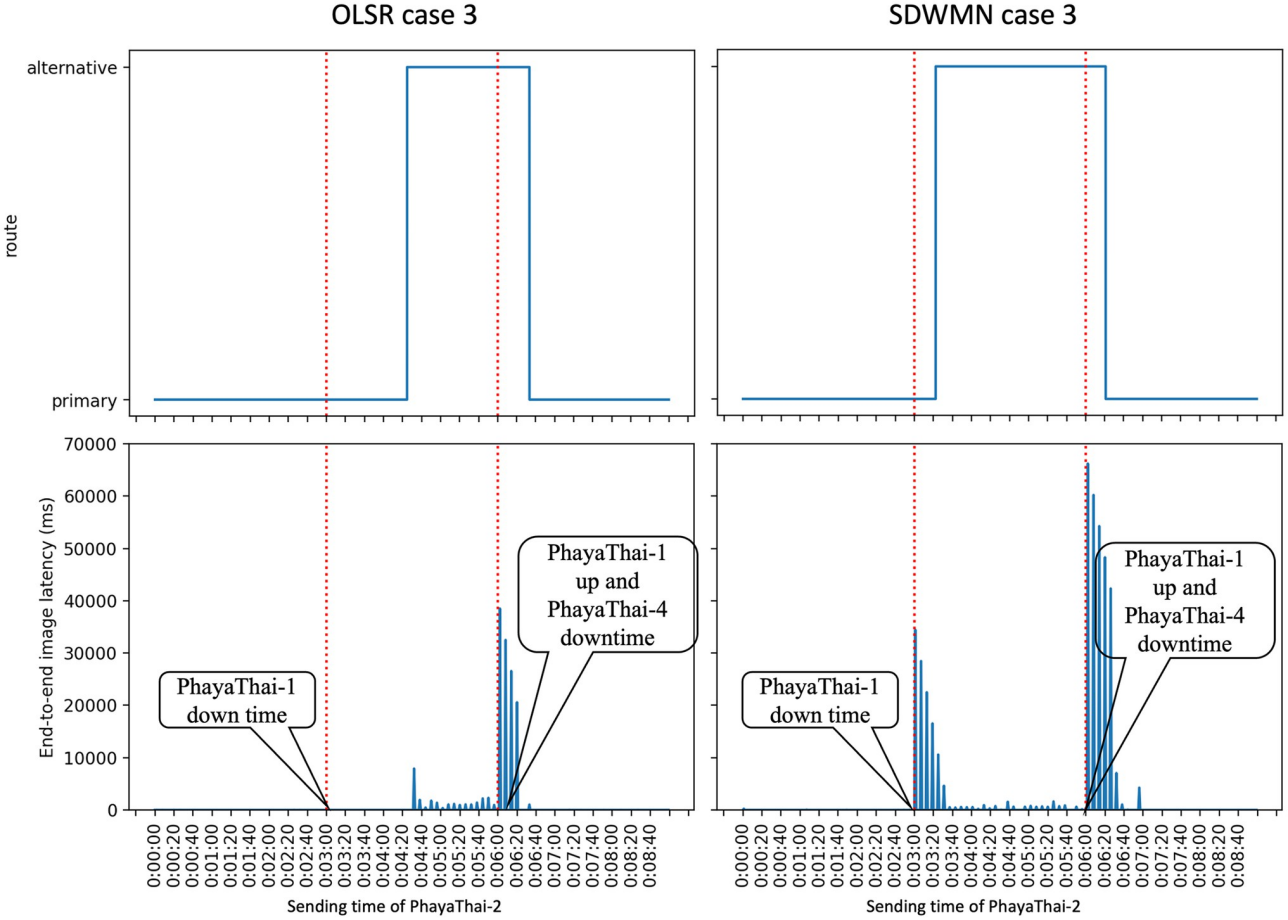
Example of case 3 for OLSR and SDWMN.

**Fig 26:**
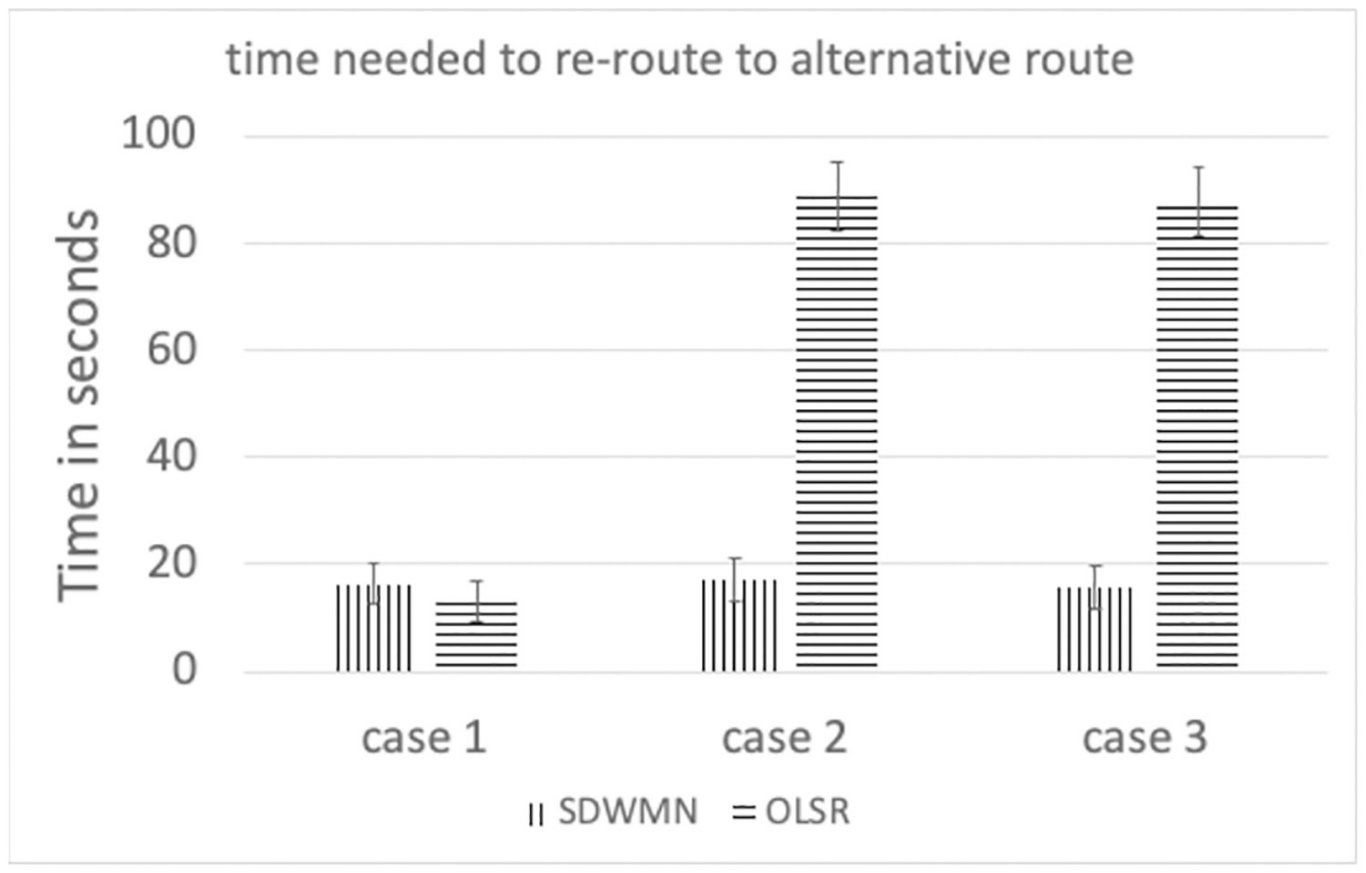
Time needed to re-route to alternative route.

**Fig 27:**
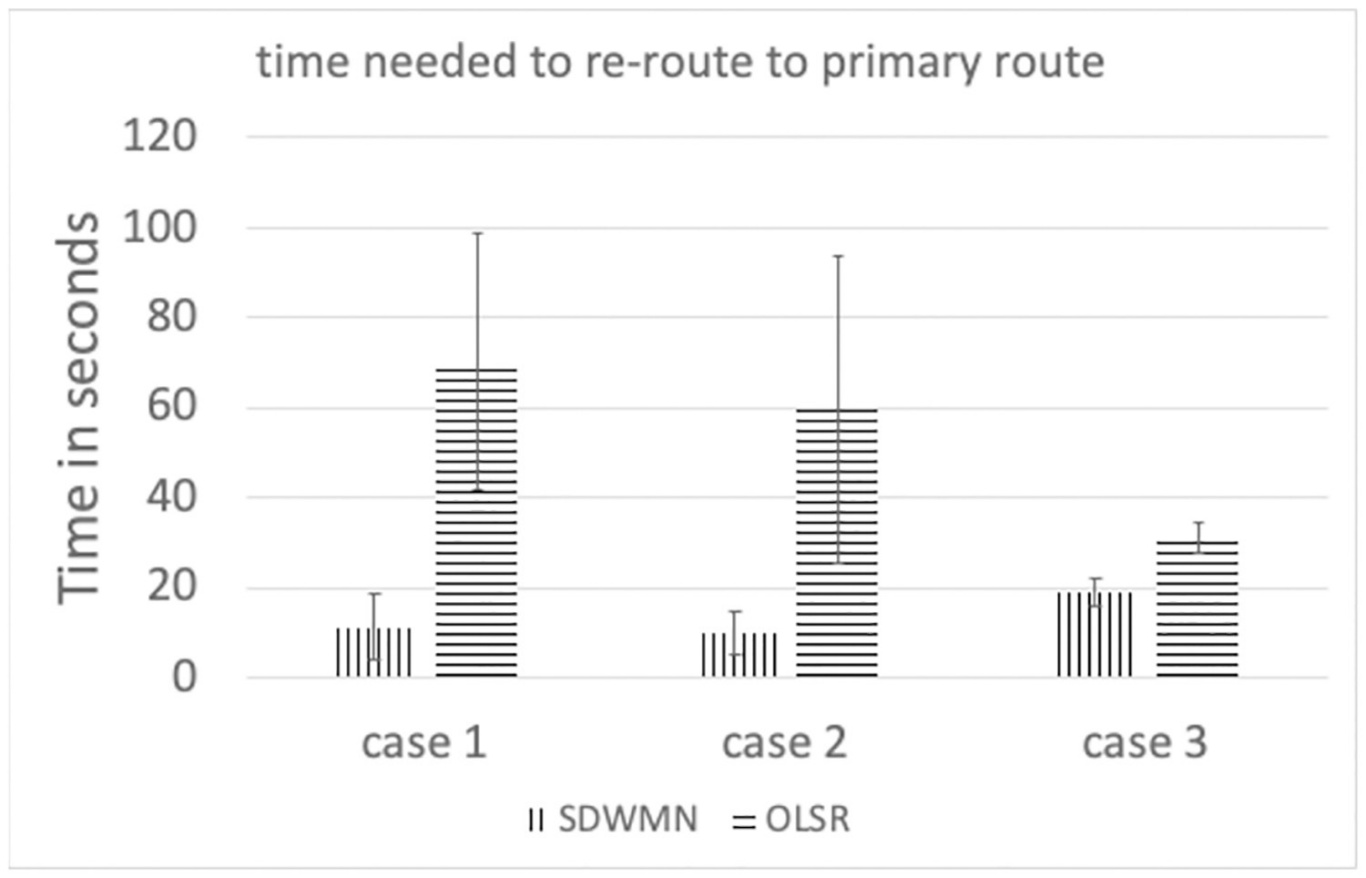
Time needed to restore to primary route.

**Fig 28:**
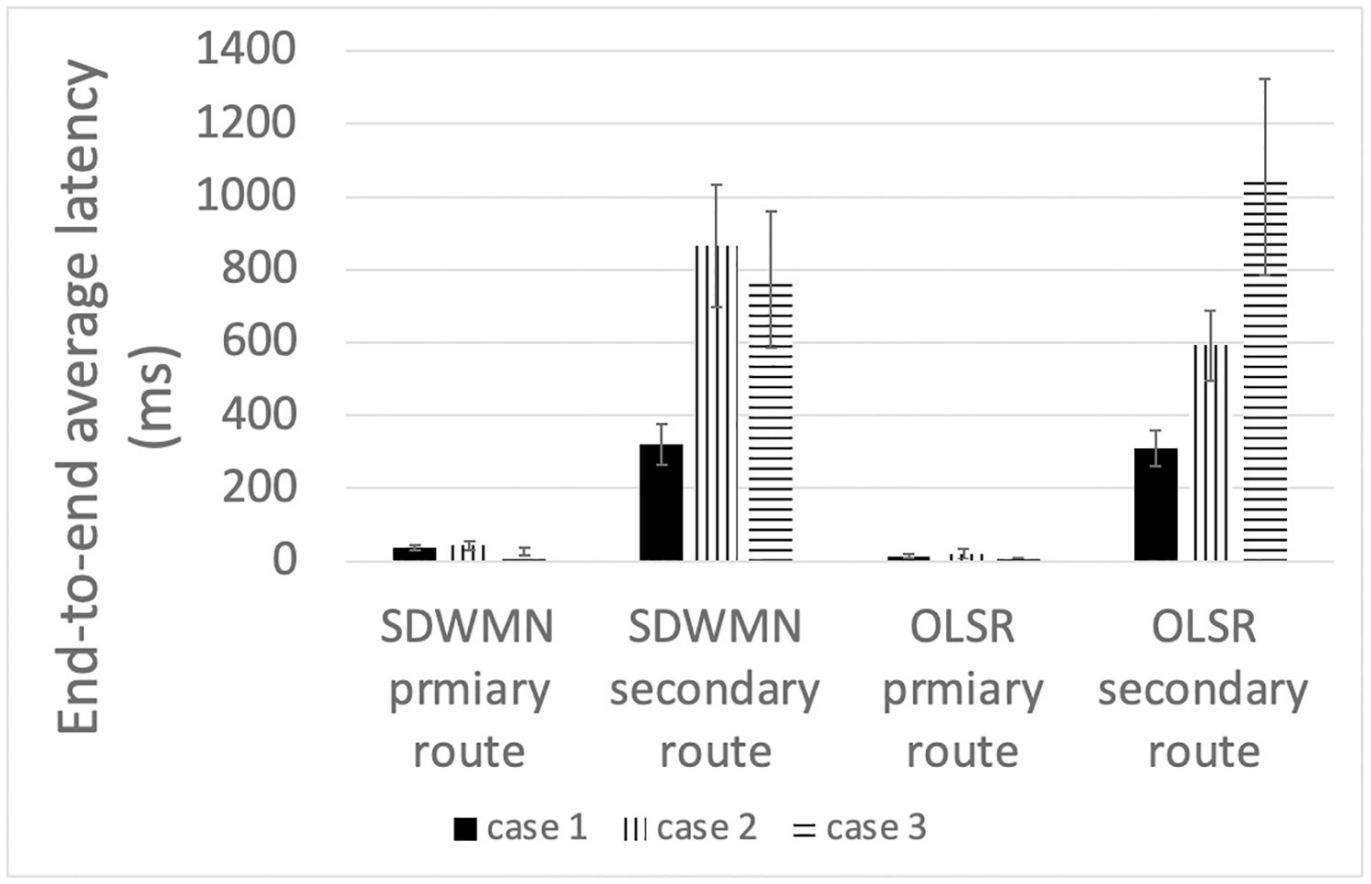
End-to-end average latency for PhayaThai-2 node for primary route and alternative route after re-routing/restoration completion.

Figs 23–25 show the example of each case for OLSR underlay network and SDWMN underlay network. In each figure, the experiment running time is 9 minutes for each test. Each test has been performed for 5 times and the final result is concluded at Figs 26 and 27 and the average end-to-end image latency in ms with 95-percent confidence intervals is shown in Fig 28.

As expected, mesh nodes running OLSR and SDWMN network need time to detect the failure of the primary route and re-route to the alternative route. Therefore, the image latency is increased when the produced image sequence from PhayaThai-2 is re-routed to the alternative route. SDWMN needs a similar amount of time to assign the flow rule from the Ryu controller to other up-running mesh nodes for re-routing to the alternative route. For the OLSR case, the HELLO message is broadcasted to the neighboring nodes to re-select the multi-point relay (MPR) node. For case 1, the maximum throughput limitation of PhayaThai-4 is set to 100 kbps. Therefore, the HELLO messages can be broadcasted faster than in case 2 and case 3 because of different throughput limitation values. The result is that the time needed for re-routing to the alternative route is less than the SDWMN case. For case 2 and case 3, the maximum throughput limitation of PhayaThai-4 is set to 50 kbps. Image sequence streaming also uses the same interface as ICMP packet sending. The throughput of PhayaThai-4 is limited at 50 kbps. Therefore, the bandwidth left for HELLO message broadcasting is less than in case 1. This results in much more time than SDWMN case 2 and case 3 for re-routing to the alternative route.

For restoring to the primary route when the PhayaThai-1 interface is recovered, SDWMN needs much less time than OLSR for every case. This is because the time for the flow rule assigned by the Ryu controller is set to 10 seconds. After 10 seconds, the SDN controller knows of the already restored node status, and hence PhayaThai-2 node path can immediately from the central SDN controller be restored to the default route, which is the primary route. However, for case 3, more control messages are needed to be transmitted to check the node reachability than in case 1 and case 2. This results in more time than case 1 and case 2 to restore the primary route. For the OLSR cases, the MPR node must be re-selected to be PhayaThai-1 to restore the primary route. If PhayaThai-4 can offer enough throughput for the data transmission, then PhayaThai-2 will keep the PhayaThai-4 as the MPR for a certain time. When the maximum throughput limit is reduced, the restoration time will also be reduced. Case 3 is the best case for restoring the primary route because PhayaThai-4 interface is shut down when PhayaThai-1 is recovered. Even in the best case for the OLSR, the restoration time is still higher than most of the SDWMN cases, due to the lack of central controllability of OLSR.

Since the buffer memory size of the Kafka producer is set to 1 MBytes, the unsent image messages are lost if there is no route to go to the destination, here local broker 1. Therefore, in some cases shown in Figs 23–25, the image message losses can be noticeable particularly right after the first failure event. Here, immediately after the failure, one can notice that there is no incoming image message until the image with large end-to-end latency values has started to re-arrive at local broker 1. For the end-to-end average latency, the OLSR can provide better performance than the SDWMN network in this particular small-scale network. This is because of the processing delay for the OVS and the in-band SDWMN control message overhead. However, such end-to-end average latency difference is marginal, i.e. less than 300 milliseconds as seen in Fig 28 for all routes. Further, this performance gap is at the best network-size scenario for OLSR protocol. The OLSR’s HELLO message packet size depends on the network size. For this small-scale network, the HELLO message packet is smaller than the SDWMN control messages. and that is why, for small-scale network experiment in this paper, the resultant latency of the standard OLSR case is better than that of the proposed SDWMN case. However, for large scale networks, the HELLO message packet size is increased quickly because each HELLO message includes the neighboring nodes’ information, which results in the nominal payload size of these HELLO messages in the order of *n*(*n* − 1) which equates to *O*(*n*^2^), where *n* is the number of nodes in the network. In OLSR, the number of hops per route using a minimum spanning tree for those messages is *O*(log*n*) and therefore, the complexity of OLSR routing overhead becomes *O*(*n*^2^ log*n*). However, for the SDWMN underlay network, control messages are sent from the SDN controller directly to the wireless mesh nodes. The number of transmissions for assigning the flow rule is *O*(*n*) to reach all the *n* nodes with the payload size per message carrying only the information of each target node (and hence requiring the space complexity in *O*(1)). And recall the deepest route from the central SDN controller for the proposed in-band SDWMN approach is simply upper-bounded by the network’s longest route dimension i.e. with *O*(*n*). So we deduce that the number of hops per route is also *O*(*n*). Therefore, the complexity of SDWMN routing overhead is *O*(*n*^2^). The conventional OLSR routing overhead becomes higher than that of our proposed SDWMN as the number of nodes *n* representing the scale of the network increases.

Based on that theoretical analysis, the comparison of this small-scale network can provide a better advantage for the OLSR protocol than the SDWMN network. Even in such worst-network scenario for SDWMN, the large control message traffic for the SDWMN results in only marginally increased end-to-end latency than that of OLSR. Also, due to the SDN central controllability, the re-routing and restoration times of the proposed system using SDWMN are significantly smaller than that of OLSR. The experimental results herein reported thus suggest the practical applicability of the proposed system.

## Conclusion and future work

In this work, we have designed and implemented the prototype for near real-time wireless image sequence streaming cloud with Apache Kafka for road traffic monitoring application for small-scale network at the target road network. This system is designed to be cost-effective by considering systematic options to reduce operational and implementation costs. The proposed design can have the fault-tolerant capability in the data flow at the level deliverable by the Apache Kafka framework. This is in addition to the re-routing capability provided by the underlying SDWMN proposed in the earlier work [20]. The testing for monitoring application performance has been investigated for the end-to-end average latency and image loss percentage by running the 13 hours of testing time from 5:40 P.M. 17^th^ November 2020 to 6:40 A.M. 18^th^ November 2020. Based on the result of the practical outdoor testbed, the end-to-end average latency and image loss percentage are within the acceptable condition, which is less than 5 seconds on average with around 10% image losses. The proposed system has also been compared with the traditional ad-hoc network, running the OLSR-based network layer, in terms of the rerouting time, restoration time and end-to-end average latency. Based on the emulated wireless network in controllable laboratory environments, the proposed SDWMN-based system outperforms the conventional OLSR-based system with potentially faster rerouting/restoration time due to SDN central controllability and with only marginally increased end-to-end average latency after re-routing/restoration completion. An algorithm complexity analysis has also been given for both systems. The overhead complexity of the SDWMN used in the proposed system is *O*(*n*^2^), which is smaller than that required by OLSR at *O*(*n*^2^ log*n*), where *n* is the number of nodes in the network. Both the experimental and complexity analysis results thus suggest the practical applicability of the proposed system. Given this promising result, it is recommended as the future research in further developing from the prototype design into the actual deployment for daily traffic monitoring operations.
